# Novel PCR detection of CRISPR/Cas systems in *Pseudomonas aeruginosa* and its correlation with antibiotic resistance

**DOI:** 10.1007/s00253-022-12144-1

**Published:** 2022-09-30

**Authors:** Mai Soliman, Heba Shehta Said, Mohammed El-Mowafy, Rasha Barwa

**Affiliations:** Department of Microbiology and Immunology, Faculty of Pharmacy, Mansoura University, Mansoura, 35516 Egypt

**Keywords:** *Pseudomonas aeruginosa*, CRISPR/Cas system, Multiplex–PCR, Antibiotic resistance, Biofilm formation

## Abstract

**Abstract:**

CRISPR (clustered regularly interspaced short palindromic repeats)-Cas (CRISPR-associated proteins) systems are considered as acquired immune mechanisms in Gram-positive and Gram-negative bacteria and also in archaea. They provide resistance/immunity to attacking bacteriophages or mobile genetic elements as integrative conjugative elements (ICE) as well as plasmid transformation. As an opportunistic pathogen, *Pseudomonas aeruginosa* has been held responsible for serious infections especially in hospitalized and immunocompromised patients. Three subtypes of type I CRISPR system (I-C, I-E, & I-F1) have been detected in *P. aeruginosa* genomes. In this work, *P. aeruginosa* isolates were collected from different clinical sources, and the three CRISPR/Cas subtypes (I-C, I-E, & I-F1) were detected via singleplex and multiplex PCR techniques using novel universal primers that were designed specifically in this study. CRISPR subtypes I-C, I-E, and I-F1 were detected in 10, 9, and 13 isolates, respectively. Furthermore, antimicrobial susceptibility of CRISPR/Cas-positive and negative isolates to different antibiotics and the capacity of biofilm formation were detected using disc diffusion method and tissue culture plate method, respectively. There was a significant correlation between the presence/absence of CRISPR/Cas system and both antimicrobial susceptibility to some antibiotics and biofilm-forming capacity among *P. aeruginosa* clinical isolates.

**Key points:**

• *A novel multiplex–PCR for detection of CRISPR/Cas-positive strains of P. aeruginosa.*

• *Understand the correlation between CRISPR/Cas systems and other characters of P. aeruginosa.*

• *Correlation between antimicrobial susceptibility and CRISPR systems in P. aeruginosa.*

**Supplementary Information:**

The online version contains supplementary material available at 10.1007/s00253-022-12144-1.

## Introduction

CRISPR (clustered regularly interspaced short palindromic repeats)/Cas (CRISPR-associated proteins) systems are currently the highlight of biology research. In 1987, a notable repetitive DNA sequence was identified in *E. coli* genome while searching for the genes implicated in phosphate metabolism (Ishino et al. 1987). Soon after, it was named CRISPR. Later on, similar sequences were reported in archaea and a wide variety of bacteria (Ishino et al. 2018). CRISPR/Cas system comprised a CRISPR array that includes short repeats interspaced by distinct DNA sequences called spacers. Such spacers are located among a group of short Cas genes that are responsible for the CRISPR immunity function (England et al. 2018). Therefore, CRISPR/Cas system is considered an acquired immunity mechanism that stores the memory of foreign DNA of invaders, in distinctive spacer sequences of the CRISPR arrays (Koonin and Makarova 2019).

The defense mechanism against foreign DNA via the CRISPR/Cas system includes three distinctive steps: immunization (spacer acquisition), CRISPR RNAs (crRNAs) biogenesis (expression), and target interference step (Barrangou 2013). The first stage includes the insertion of protospacers, which are pieces of foreign DNA from invading bacteriophages or plasmids into the CRISPR array. Protospacers act as a stored memory in the bacterial cell for the defense against the same plasmid or virus when it attacks the cell again. During the second stage, these spacers that are interrupted by repeats are expressed as small guide crRNAs. Lastly, in the interference stage, Cas proteins help crRNAs to quarry and damage invading bacteriophages or plasmids (England et al. 2018; Van der Oost et al. 2009).

CRISPR/Cas systems are categorized into two groups (Class I & Class II) according to variation in Cas protein structure and sequence divergence among the effector modules (Makarova et al. 2020). Both classes comprise 6 types. Class I includes 3 types (I, III, & IV) and utilizes a multi-subunit crRNA-effector complex that carries on the mechanistic function of CRISPR immunity (adaptation, expression, and target interference). On the other hand, Class II includes the types II, V, and VI that has a single subunit crRNA-effector module (Makarova et al. 2015). Three types (I, II, and V) target/damage foreign DNA only; type VI modulates RNA only; whereas type III targets both RNA and DNA (Wang et al. 2019).

CRISPR systems are less common in bacteria than archaea as they were detected in approximately 50% and 87% of bacterial and archaeal genomes, respectively (Makarova et al. 2013). Type I CRISPR system is equally distributed in both archaea and bacteria with complete single unit loci, whereas type IV and type V CRISPR systems are less common in both of them (Makarova et al. 2015). Type III CRISPR system is more profuse in archaea than bacteria, which possess type II systems chiefly (Makarova et al. 2015). CRISPR/Cas systems are present in Gram-positive and Gram-negative bacteria (Louwen et al. 2014). Further classification of these systems into subtypes has been adopted in bacteria harboring such systems. For example, in *S. pyogenes*, two CRISPR subtypes: type I-C (Class I) and II-A (Class II) were detected (Nozawa et al. 2011).

In bacteria, CRISPR/Cas system plays a substantial role by providing resistance against plasmid transformation and invading viruses like bacteriophages (Barrangou et al. 2007; Marraffini and Sontheimer 2008). CRISPR/Cas cassettes harbor various loci that can uptake spacers from invading bacteriophages and hence provide immunity against recurrent infection by the same virus. It also can obtain spacers from self-replicating plasmids carrying antibiotic resistance determinants, leading to cleavage of plasmids (Garneau et al. 2010). CRISPR/Cas systems were correlated with the expression of some virulence genes in bacteria. In *Pseudomonas aeruginosa*, for example, it was proven to be important in swarming motility used. Zone of inhibition and biofilm formation that are significant features of *P. aeruginosa* pathogenicity (Palmer and Whiteley 2011).

*P. aeruginosa* is an opportunistic microbe and a dominant cause of serious infections particularly in hospitalized and immunocompromised patients (Eladawy et al. 2021). It has been held responsible for several infections in the lungs (pneumonia), implants, and wounds, in addition to hospital-acquired infections (Lima et al. 2017). Three subtypes of type I CRISPR/Cas system were identified in the genomes of *P. aeruginosa* strains (Fig. S1): I-C, I-F1, and I-E (Cady et al. 2011; van Belkum et al. 2015). It was documented that the subtype I-F1 has the main function as an immune system, providing immunity against numerous viruses including bacteriophages (Cady et al. 2012). The standard *P. aeruginosa* strain PAO1 does not have a CRISPR/Cas system but the PA14 strain has a type I-F1 CRISPR system (Jeukens et al. 2014). Interestingly, the genomes of *P. aeruginosa*, which contain CRISPR/Cas systems, were relatively smaller than those missing CRISPR/Cas systems (van Belkum et al. 2015).

According to our knowledge, there is no established PCR-based method for detection and differentiation between the known subtypes of type I CRISPR/Cas system in *P. aeruginosa*. Therefore, our current study aims to establish a novel detection method for different subtypes of the CRISPR/Cas system in *P. aeruginosa*. Moreover, the correlation between the existence of CRISPR systems in the bacterial isolates and both the antimicrobial susceptibility to different antimicrobial agents and biofilm-forming capacity was assessed.

## Materials and methods

### Collection of clinical *P. aeruginosa* isolates

All clinical samples were collected under the approval of Research Ethics Committee (Faculty of Pharmacy, Mansoura University, Egypt) with the ethical code 2020–80. Participants in this work have signed informed consents. The collection of clinical isolates, from Mansoura hospitals, took place for 7 months (June–December 2020).

Bacterial isolates were identified as *P. aeruginosa* employing adequate microbiological laboratory techniques (Collee et al. 1996; Mackie 2006) by streaking on cetrimide agar media and the resulting colonies were examined after Gram staining under a microscope. Other confirmatory identification tests included testing for their ability to grow at 42 °C and oxidase and catalase production, in addition to the detection of the characteristic sweetish odor and green pigment after cultivation on cetrimide agar plates. For long-term storage, purified isolates were cultured in Muller Hinton Broth (MHB) medium and then preserved in the MHB containing glycerol (20% v/v) at − 80 °C. In all experiments, PAO1 was employed as a reference strain of *P. aeruginosa*.

### Design of specific primers for different subtypes of CRISPR systems

Genes’ sequences (25 sequences for each gene, Table S1) belonging to different CRISPR-Cas cassettes of reported subtypes of *P. aeruginosa* were recovered from GenBank and subjected to alignment via the multisequence alignment program (Clustal Omega) that is provided by EMBL-EBI website (Goujon et al. 2010; McWilliam et al. 2013; Sievers et al. 2011). For further analysis of aligned sequences and detection of conserved regions, the Jalview program was used (Waterhouse et al. 2009).

**Fig. 1:**
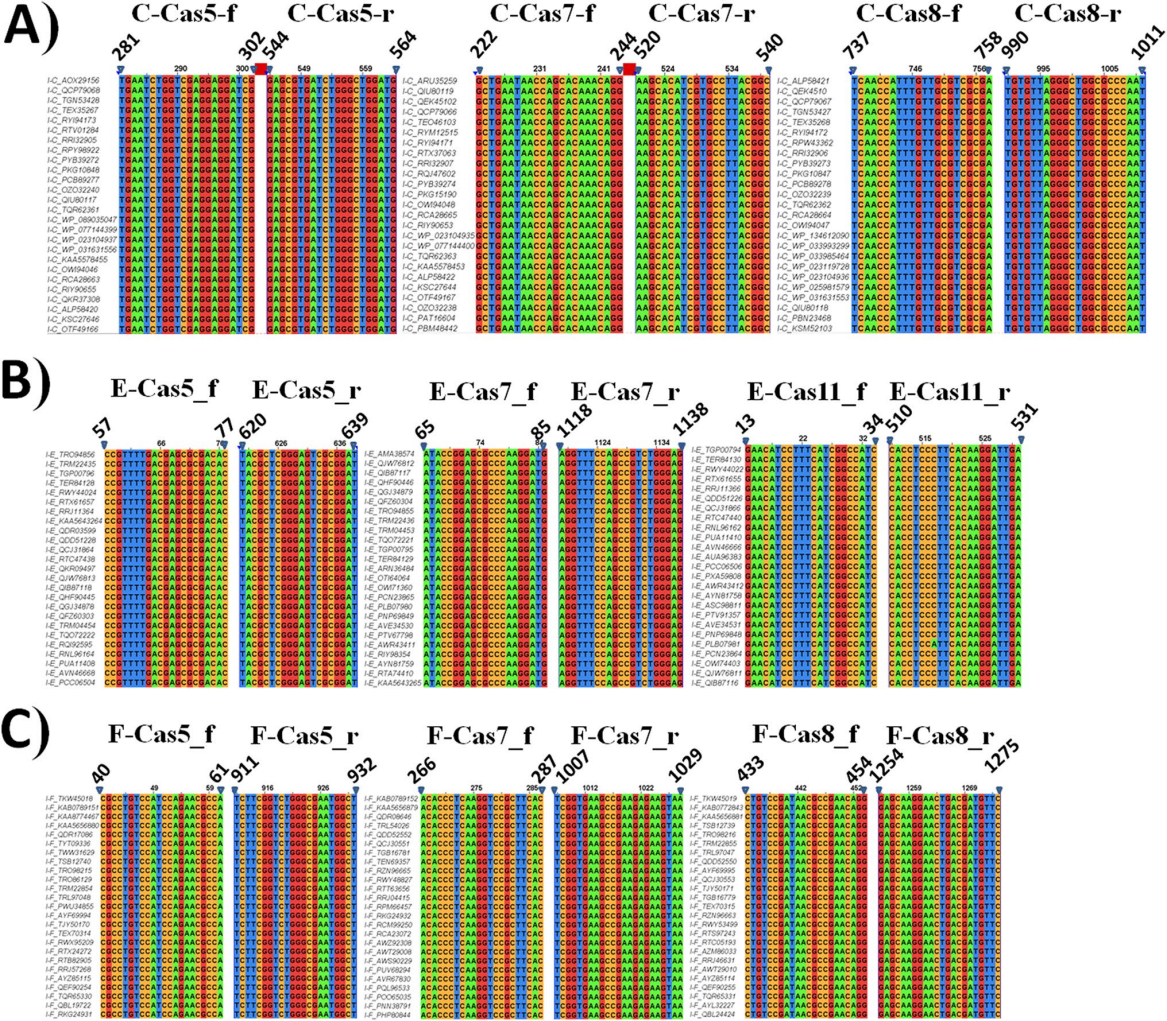
Analysis of multiplesequence alignments of 25 partial DNA sequences of *P. aeruginosa* strains in the CRISPR/Cas cassettes of different subtypes showing the target conserved regions for primers design. **A** Subtype I-C. **B** Subtype I-E. **C** Subtype I-F1. The ID of each partial sequence was named by the subtype followed by its accession number in the GenBank

Different primers were designed at different conserved regions of different genes after fulfilling the criteria of optimum GC% (40–60%) and minimal probabilities of hairpin and primer dimer formation. Later on, primers fulfilling the previous criteria were further subjected to bioinformatic analysis via blast against the whole genomes of *P. aeruginosa* species using the free blast tool provided by the National Center for Biotechnology Information. Only primers showing specific binding with *P. aeruginosa* genomes were selected for further PCR experiments. Accordingly, primer pairs were selected for the following genes in each subtype’s CRISPR/Cas cassette: *cas5*, *cas7*, and *cas8* genes for subtypes I-C and I-F1 in addition to *cas5*, *cas7*, and *cas11* genes for subtype I-E (Table 1). The alignment of the conserved regions for primer design of the previously mentioned target genes is shown in Fig. 1. Full alignments of the whole sequences of these genes are demonstrated in Fig. S2.

**Table 1:** Primers used for detection of CRISPR/Cas subtypes in *P. aeruginosa* clinical isolates

CRISPR-Cas subtype	Gene (size in bp)	Primer name_ forward (f) or reverse (r)	Sequence (5′-3′)	Binding position in the gene	Annealing temperature (°C)	Amplicon size (bp)	Primer design
I-C	*cas7* (870)	C-Cas7_f	GCTGAATAACCAGCACAAACAGG	222–244	53	318	This study
		C-Cas7_r	GCCGTAAGGCACGATGTGCTT	520–540	53		
	*cas8* (1794)	C-Cas8-f	TCAACCATTTGTTGCGTCGCGA	737–758	53	274	This study
		C-Cas8-r	ATTGGGCGCCAGCCCTAACACA	990–1011	53		
	*cas5* (675)	C-Cas5_f	TGAATCTGGTCGAGGAGGATCG	281–302	53	283	This study
		C-Cas5_r	CATCCAGCCCAGATCACGCTC	544–564	53		
I-E	*cas5* (662)	E-Cas5-f	CCGTTTTGACGAGCGCGACAC	57–77	53	582	This study
		E-Cas5-r	ATCCGCGACTCCCGAGCGTA	620–639	53		
	*cas7* (1142)	E-Cas7_f	ATACCGGAGCGCCCAAGGATG	65–85	54	1073	This study
		E-Cas7_r	CTCCCAGACGGCTGGAAACCT	1118–1138	54		
	*cas11* (531)	E-Cas11_f	GAACATCCTTTCATCGGCCATC	13–34	53	518	This study
		E-Cas11_r	TCAATCCTTGTGAAGGGAGGTG	510–531	53		
I-F1	*cas7* (1029)	F-Cas7_f	ACACCCTCAAGGTCCGCTTCAC	266–287	55	763	This study
		F-Cas7_r	TTACTTCTCTTCGGCTTCACCGA	1007–1029	55		
	*cas5* (984)	F-Cas5_f	CGCCTGTCCATCCAGAACGCCA	40–61	55	829	This study
		F-Cas5_r	AGCCATTCGCCCAGACCGAAGA	911–932	55		
	*cas8* (1305)	F-Cas8_f	CTGTCCGATAACGCCGAACAGG	433–454	55	842	This study
		F-Cas8_r	GAACATCGTCAGTTCCTTGCTC	1254–1275	55		

bp: base pair

### Singleplex and multiplex PCR detection of CRISPR/Cas subtypes in *P. aeruginosa*

The whole genome extracts of different isolates of *P. aeruginosa* were extracted by colony PCR as described previously (Eladawy et al. 2021; Englen and Kelley 2000). Briefly, isolates were cultured in MHB overnight and then streaked on cetrimide agar plates. A single separate colony from each isolate was suspended in nuclease-free water (50 μl) and boiled for 10 min. The suspension of the heat-lysed cells was centrifuged, and the supernatant (containing DNA extract) was kept at − 80 °C till further investigation.

Each of the three selected genes of each CRISPR/Cas subtype in *P. aeruginosa* was detected by conventional singleplex PCR in the collected isolates using the designed primer pairs (Table 1). Each PCR reaction mixture included the following: 10 μl of 2 × Dream Taq™ Green PCR Master Mix (Thermo Scientific, USA), 0.8 μl of forward/reverse primers each (10 μM), DNA extract (volume containing 50 ng), and finally the mixture volume was adjusted to 20 μl. A negative control tube was incorporated in all PCR experiments using DNA extract of PAO1 strain. PCR conditions comprised an initial step of denaturation (95 °C/5 min), then 35 cycles of denaturation (95 °C/30 s), annealing (specific temperature for pairs of primers as demonstrated in Table 1/30 s), and extension (72 °C/60 s). At last, the reaction was concluded by a final extension (72 °C/5 min). Successful amplification of target PCR products, according to the amplicons’ size in Table 1, was assured by agarose gel electrophoresis employing 1.5% gels containing ethidium bromide followed by visualization using a gel documentation system (Acculab).

Multiplex PCR technique was implemented for the detection of the CRISPR/Cas subtypes of *P. aeruginosa* in a single reaction tube. For such purpose, 3 strategies were followed in our study.

- The 1^st^ strategy: included the use of a mixture of primer pairs (Mix1) that are designed for amplification of *cas5* gene in the three different subtypes; I-C (amplicon size: 283 bp), I-E (amplicon size: 582 bp), and I-F1 (amplicon size: 829 bp).
- The 2^nd^ strategy: a mixture of primer pairs (Mix2) that are used for amplification of *cas7* gene in the different three subtypes; I-C (amplicon size: 318 bp), I-E (amplicon size: 1073 bp), and I-F1 (amplicon size: 763 bp) were used.
- The 3^rd^ strategy: primer pairs’ mixture (Mix3) for the amplification of *cas8* gene (subtypes I-C and I-F1) and *cas11* (subtype I-E) with expected amplicon sizes of 274, 842, and 518 bp for *cas8* (subtype I-C), *cas8* (subtype I-F1), and *cas11* (subtype I-E), respectively.

The efficiency of the three strategies was evaluated in randomly selected CRISPR/Cas-positive isolates (3 positive isolates from each subtype).

### Sequencing

A representative isolate from each CRISPR/Cas subtype was selected for sequencing of the three target genes of each subtype in this study. For such step, targeted genes were amplified employing Phusion High-Fidelity DNA Polymerase (Thermo Scientific, Scientific Inc.) and utilizing the specified primers in Table 1. PCR reactions were prepared based on the manufacturer’s recommendations. Purification of the target amplicons was performed via a gel extraction kit (Qiagen, Hilden, Germany). After purification, amplicons were shipped to Sigma Scientific Service Technical Support (Cairo, Egypt) for sequencing using Applied Biosystems 3500 XL Genetic Analyzer employing the reverse primers designed for each target gene. The chromatograms of the partial DNA sequences of the target genes were visualized and analyzed using the FinchTV program.

### Antimicrobial sensitivity of bacterial isolates

Antimicrobial susceptibility pattern of the 32 CRISPR/Cas-positive *P. aeruginosa* isolates and 32 randomly selected CRISPR/Cas-negative isolates from the same sources was conducted using the Kirby-Bauer disc diffusion technique (Bauer 1966). Susceptibility to various groups of antimicrobial agents was examined; therefore, antibiotic discs (Bioanalyse®, Turkey): piperacillin (100 μg), piperacillin-tazobactam (100/10 μg), ceftazidime (30 μg), cefepime (30 μg), amikacin (30 μg), imipenem (10 μg), meropenem (10 μg), ciprofloxacin (5 μg), and levofloxacin (5 μg) were used. Zone of inhibition diameter was determined and elucidated in accordance with Clinical and Laboratory Standards Institute guidelines (CLSI 2021).

### Biofilm detection by tissue culture plate method

The capacity of biofilm formation among the 32 CRISPR/Cas-positive *P. aeruginosa* isolates and the randomly selected 32 CRISPR/Cas-negative isolates was examined in vitro using the tissue culture plate method under static conditions as previously described (Stepanović et al. 2000). In brief, isolates were cultured in tryptic soy broth complemented with 1% anhydrous glucose (TSBG) and incubated at 37 °C overnight. Using the TSBG medium, bacterial cultures were adjusted at 600 nm to 0.2. Then, the adjusted cultures were transferred to a 96-well microtitre plate (100 μl/well, 4 wells/isolate). Following overnight incubation at 37 °C, bacterial cultures (from every well) were gently withdrawn and the microtitre plate was rinsed 3 times with phosphate-buffered saline (PBS, 200 μl/well) to discard non-adherent cells. The plate was dried in air, followed by adding 150 μl absolute methanol for fixation of the adherent cells. After the aspiration of methanol, the adhered biofilm was stained using 1% crystal violet (150 μl/well) for 20 min. To remove excess dye, the plates were washed gently three times using distilled water then the plates were kept inverted in the air until dry. Approximately 150 μl of glacial acetic acid (33% V/V) was transferred to each well for solubilization of the stained biofilm. Then, OD (at 540 nm) was assessed by means of ELx808™ Absorbance Microplate Reader (BioTek Instruments Inc., Winooski, VT). Negative control of medium only was included in each experiment (Di Domenico et al. 2016; Eladawy et al. 2020; Perez et al. 2011).

To evaluate the biofilm-forming capacity of *P. aeruginosa* isolates, the mean optical density (ODi) of the bacterial isolate was determined. A cutoff value (ODC) was estimated as three standard deviations over the negative control’s mean OD. Isolates were considered non-biofilm producers (ODi ≤ ODC), weak producers (ODC < ODi ≤ 2 ODC), moderate producers (2 ODC < ODi ≤ 4 ODC), and strong producers when (4 ODC < ODi) (Lima et al. 2017; Stepanović et al. 2000).

### Statistical analysis

Comparison of frequencies of distribution of CRISPR/Cas system and its subtypes, antimicrobial susceptibility, and biofilm formation capacity among *P. aeuginosa* isolates were evaluated via chi-square test or Fisher’s exact test (*P* < 0.05). Data was assessed via SPSS software (version 20.0; SPSS, Chicago, IL, USA).

## Results

### Isolation and identification of *P. aeruginosa*

In our work, 122 isolates of *P. aeruginosa* were collected from Mansoura hospitals for 7 months (June–December 2020). *P. aeruginosa* isolates were recovered from diverse clinical resources including: urinary tract infections (60 isolates), respiratory tract infections (13 isolates), wound exudate (24 isolates), blood (15 isolates), and other sources (10 isolates) like contact lenses, burns, and vaginal swabs samples (Table S2).

### Singleplex and multiplex PCR detection of CRISPR/Cas subtypes in *P. aeruginosa*

**Fig. 2:**
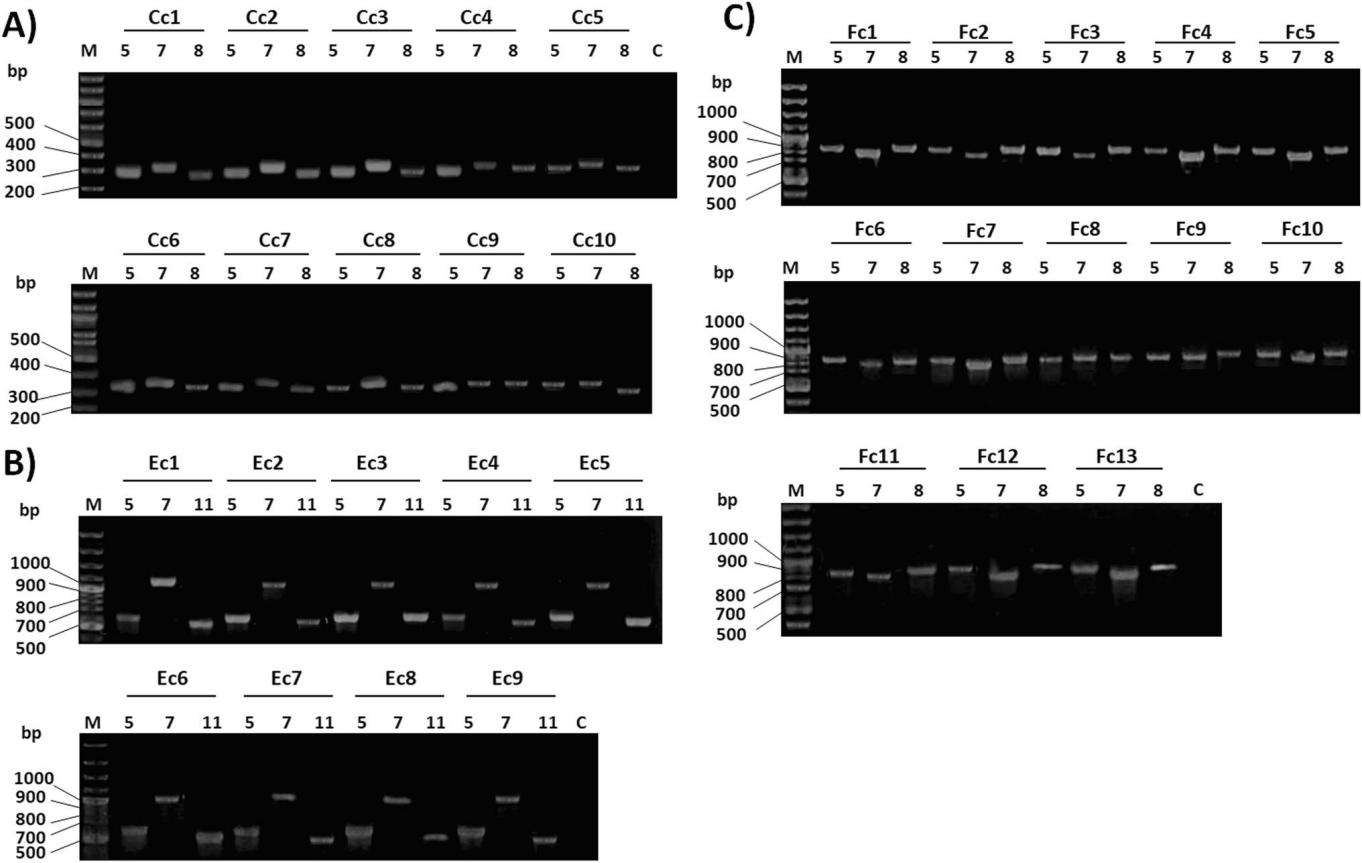
Detection of *cas* genes of different subtypes of CRISPR/Cas systems in *P. aeruginosa* clinical isolates. Lane M: DNA ladder (100 bp ladder plus SM0321). Lane C: negative control (PAO1 strain). Lanes 5, 7, 8, 11: refer to the detection of *cas5*, *cas7*, *cas8*, and *cas11* respectively. **A** Detection of *cas5* (283 bp), *cas7* (318 bp), and *cas8* (274 bp) genes in 10 clinical isolates of subtype I-C. **B** Detection of *cas5* (582 bp), *cas7* (1073 bp), and *cas11* (518 bp) genes in 9 clinical isolates of subtype I-E. **C** Detection of *cas5* (829 bp), *cas7* (763 bp), and *cas8* (842 bp) genes in 13 clinical isolates of subtype I-F1

Conventional singleplex PCR indicated that 32 isolates harbored CRISPR/Cas systems. CRISPR/Cas-positive isolates were named using a capital letter referring to the detected subtype (C, E, or F1) followed by “c” (for clinical isolate) and lastly the number of the isolate, whereas CRISPR/Cas-negative isolates were given the name Nc tracked with the number of the isolate as mentioned in Table S2. CRISPR/Cas subtype I-C was detected in 10 isolates (Fig. 2A), while 9 isolates contained the subtype I-E (Fig. 2B) and 13 isolates harbored the subtype I-F1 (Fig. 2C). Each of the three selected genes (*cas5*, c*as7*, and *cas8*) of CRISPR/Cas subtypes I-C and I-F1 was detected in a separate PCR reaction for each isolate as shown in Fig. 2A and 2C. The *cas5*, *cas7*, and *Cas11* genes were detected in each isolate harboring the CRISPR/Cas subtype I-E as demonstrated in Fig. 2B.

**Fig. 3:**
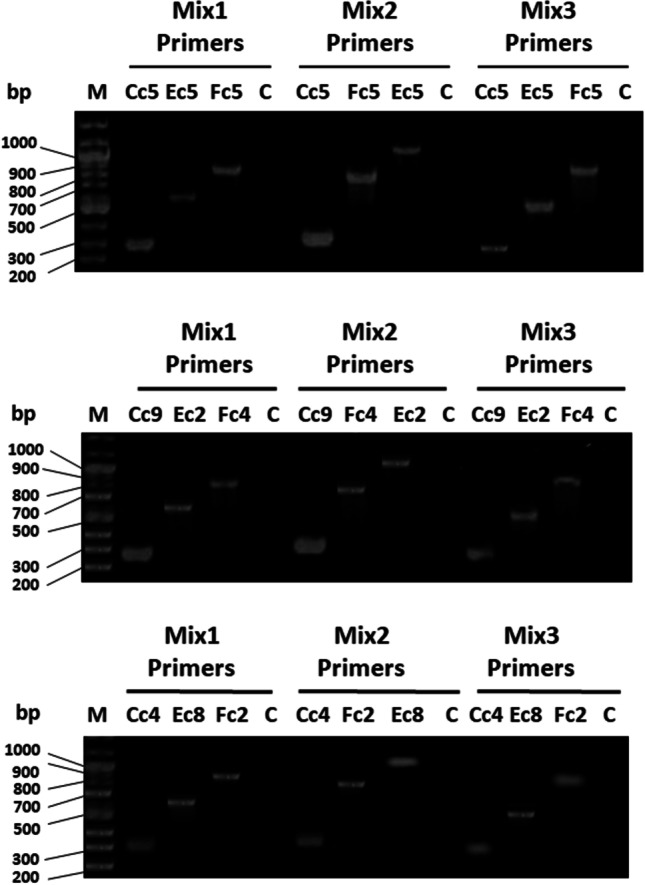
Agarose gel electrophoresis for *cas5*, *cas7*, and *cas8/cas11* genes of 9 randomly selected CRISPR/Cas-positive clinical isolates (3 positive isolates from each subtype). Lane M: DNA ladder (100 bp ladder plus). Lane C: negative control (PAO1 strain) with no CRISPR-Cas subtype), bp: base pair. For Mix1 primers: amplicons’ size of *cas5* genes in subtypes I-C, I-E, and I-F1 are 283, 582, and 829 bp, respectively. For Mix2 primers: amplicons’ size of *cas7* genes in subtypes I-C, I-F1, and I-E are 318, 763, and 1073 bp, respectively. For Mix3 primers: amplicons’ size of *cas8* genes in subtypes I-C and I-F1 are 274 and 842 bp respectively and that of *cas11* gene in subtype I-E is 518 bp

Multiplex PCR was evaluated for its efficiency to detect the CRISPR/Cas subtype of *P. aeruginosa* in a single reaction. This was performed for all CRISPR/Cas-positive isolates using the primer mixtures: Mix1, Mix2, and Mix3. All primer mixtures specifically detected the CRISPR/Cas subtype, as determined in singleplex PCR, and representative samples are shown in Fig. 3. In multiplex PCR, CRISPR/Cas-negative strain (PAO1) was also included in the multiplex PCR experiments and did not show any interference with any of the adopted primer mixtures (Fig. 3).

### Sequencing

The obtained partial sequences of the amplified genes in the CRISPR/Cas subtypes I-C, I-E, and I-F1 were successfully identified as the corresponding expected genes (100% identity) using the BLASTN search, on the NCBI website, against *P. aeruginosa* genome. Accordingly, the obtained sequences were uploaded to DDBJ/EMBL/GenBank database. The partial sequences of *cas5*, *cas7*, and *cas8* genes in the isolates Cc5, Cc9, and Cc4 (subtype I-C) were given the accession numbers LC685202, LC685203, and LC685204, respectively, while those of *cas5*, *cas7*, and *cas8* genes in the isolates Fc4, Fc2, and Fc5 (subtype I-F1) got the accession numbers LC685205, LC685206, and LC685207, receptively. Finally, the partial sequences of *cas5*, *cas7*, and *cas11* genes in the isolates Ec5, Ec8, and Ec2 (subtype I-E) were given the accession numbers LC685208, LC685209, and LC685210, respectively.

### Antimicrobial sensitivity of bacterial isolates

The highest percentage of antibiotic resistance among CRISPR/Cas-positive isolates was observed for ceftazidime (100%) followed by cefepime (96.9%). While, the lowest percentage of resistance was detected against amikacin (18.7%), ciprofloxacin, and levofloxacin (21.9% each). Furthermore, the highest percentage of antibiotic resistance among CRISPR/Cas-negative isolates was noticed with ceftazidime (84.4%) followed by piperacillin (78.1%). While the lowest percent was observed with piperacillin-tazobactam (18.7%) and amikacin (21.9%) as shown in Table 2 and Table S2.

**Table 2:**
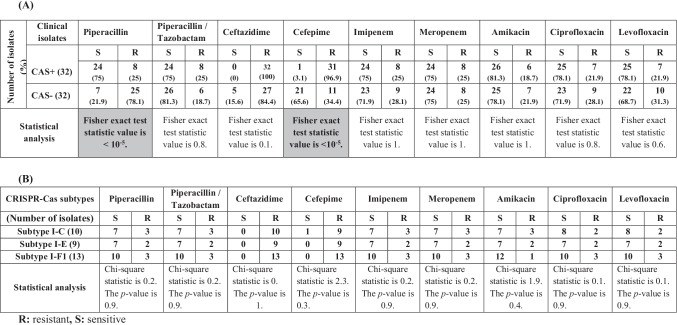
Correlation between antimicrobial susceptibility of *P. aeruginosa* clinical isolates and the presence of CRISPR/Cas system (A) and its subtypes (B)

Statistical analysis indicated a significant correlation (*P* < 0.05) between the susceptibility of clinical isolates to piperacillin and the presence/lack of the CRISPR/Cas system (Table 2). Higher sensitivity to piperacillin was observed among CRISPR/Cas-positive isolates (75%) in comparison with CRISPR/Cas-negative ones (21.9%). Moreover, the use of tazobactam in combination with piperacillin did not affect the sensitivity among CRISPR/Cas-positive strains while it increased the sensitivity among CRISPR/Cas-negative isolates (81.3%). There is no significant correlation between CRISPR/Cas subtypes and susceptibility to different antimicrobials tested (Table 2).

### Biofilm detection by TCP method

CRISPR/Cas-positive *P. aeruginosa* clinical isolates were categorized according to biofilm formation capacity into strong producers (18.8%), moderate producers (40.6%), and weak producers (25%). A low percent of these isolates did not produce biofilm (15.6%). In contrast among CRISPR/Cas-negative isolates, the highest percent were weak and non-biofilm producers (37.5% each), the lowest percent of isolates were strong producers about 9.4%, and 15.6% of the isolates were moderate producers (Table 3 and Table S2).

**Table 3:**
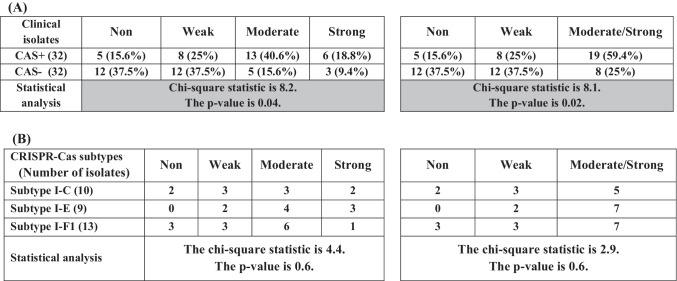
Correlation between biofilm formation capacity of *P. aeruginosa* clinical isolates and the presence of CRISPR/Cas system (A) and its subtypes (B)

Statistical analysis indicated a significant correlation (*P* < 0.05) between biofilm-forming capacity and the existence of CRISPR systems in clinical isolates of *P. aeruginosa* (Table 3). Most of the CRISPR/Cas-positive strains were strong/moderate biofilm producers in contrast with CRISPR/Cas-negative isolates that were weak or non-biofilm producers. CRISPR/Cas subtypes did not have a significant correlation with biofilm formation. Biofilm-forming capacity was nearly the same in the three subtypes (Table 3).

## Discussion

Studies that detected CRISPR/Cas systems in isolates of *P. aeruginosa* utilized computational approaches such as CRISPRCasFinder to detect CRISPR subtypes in sequenced genomes via Illumina sequencing (Silveira et al. 2020; van Belkum et al. 2015; Wheatley and MacLean 2021). CRISPRCasFinder is a software program that can be used for the prediction of CRISPR arrays and their associated proteins (Couvin et al. 2018). To our knowledge, there are no specific primers that can exclusively detect CRISPR/Cas systems and their subtypes in *P. aeruginosa* via PCR. Therefore, we were interested in designing specific primers for the detection of known CRISPR systems in *P. aeruginosa* via traditional PCR rather than expensive techniques of complete genome sequencing.

Not all strains of *P. aeruginosa* species harbor CRISPR systems (van Belkum et al. 2015). Type I system, to which the three common subtypes in *P. aeruginosa* belong, can be distinguished from other types by its signature gene: *cas3* (Makarova et al. 2015). However, the selection of *cas3* gene as a target for PCR detection of CRISPR/Cas-positive strains of *P. aeruginosa* will be hindered by its high similarity to other genes, which are not related to CRISPR/Cas system as helicases (Makarova et al. 2015, 2020).

In *P. aeruginosa*, subtypes I-C, I-E, and I-F1 include 7, 8, and 7 cas genes respectively (Fig. S1) (Makarova et al. 2020). Type I belongs to class I, whose subtypes can be classified according to sequence similarity clustering of effector proteins (Fig. S1) (Makarova et al. 2015). Interestingly, there is a tremendous difference in those proteins even within each subtype (Makarova et al. 2015). Therefore, genes in the effector complex of each subtype were selected as a target for PCR detection and were investigated for the presence of conserved regions after the alignment of 25 sequences for each gene. Moreover, the selected primers in the conserved regions were blasted against *P. aeruginosa* genomes to exclude those showing no specific binding elsewhere in the genome. Finally, we adopted in this work primers in the conserved regions of the following genes: *cas5*, *cas7*, and *cas8* genes in the subtypes I-C and I-F1, in addition to *cas5*, *cas7*, and *cas11* genes in the subtype I-E.

It should be mentioned that we could not design a primer pair that can work for the same effector protein in the three known subtypes of CRISPR systems of *P. aeruginosa*. The reason for this is that we could not find any conserved regions for the selection of primers in the aligned sequences of all the subtypes harboring *cas5*, *cas7*, *and cas8*.

After the successful detection of the selected gene of each CRISPR/Cas subtype in the CRISPR/Cas-positive isolates via conventional single PCR (Fig. 2), we were motivated to identify CRISPR/Cas-positive strains and further detect its subtype in a single reaction via multiplex PCR. For such purpose, we kept in mind, initially when selecting final primer pairs for singleplex PCR detection of genes of each subtype, that the resulting amplicons would differ in size to the extent that they can be resolved efficiently by agarose gel electrophoresis when used in mixtures for multiplex PCR. Therefore, it is clearly obvious that any of the primers’ mixture (Mix1, Mix2, or Mix3) would precisely and specifically detect CRISPR/Cas-positive isolates and also its subtype in a single-step reaction and without interference from *PAO1* strain (CRISPR/Cas-negative strain) (Fig. 3).

**Fig. 4:**
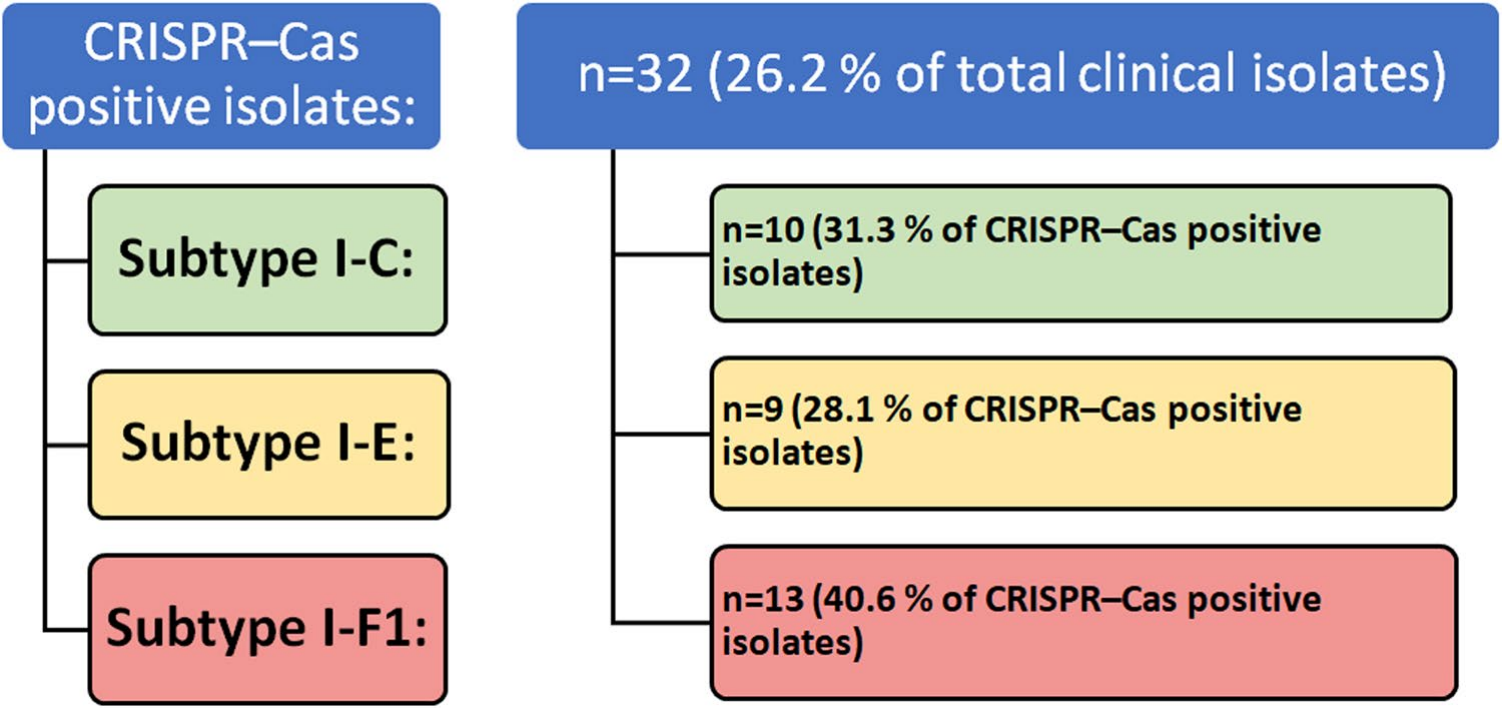
Number (*n*) and percent of PCR-detected CRISPR/Cas-positive isolates and their subtypes

CRISPR systems were detected in 26.2% of the collected clinical isolates. In positive CRISPR/Cas subtypes, the most predominant subtype was I-F1 (40.6%), then I-C (31.3%), and lastly I-E (28.1%). van Belkum et al. (2015) indicated that the subtypes I-E and I-F1 are the most common subtypes found in *P. aeruginosa*. However, in the last study, the authors identified the subtype I-C for the first time; therefore, it is expected that its rate of detection would increase in the proceeding studies. A summary of the detected subtypes in this work is illustrated in the schematic diagram of Fig. 4

An elevated level of resistance to β-lactam antibiotics was detected among *P. aeruginosa* isolates clinically recovered from diverse sources in Mansoura hospitals, Egypt. Carbapenems, fluoroquinolones, or aminoglycosides could be employed for the management of infections caused by β-lactam-resistant isolates. Our results have indicated higher resistance to piperacillin among CRISPR/Cas-negative strains compared to CRISPR/Cas-positive ones (Table 2). Moreover, the use of tazobactam in combination with piperacillin did not affect the sensitivity among CRISPR/Cas-positive strains while it increased the sensitivity among CRISPR/Cas-negative isolates indicating the production of serine β-lactamases among CRISPR/Cas-negative-resistant isolates that are capable of hydrolyzing piperacillin that could be acquired through horizontal gene transfer (HGT).

*P. aeruginosa* could develop resistance to various groups of antimicrobial agents through HGT or modification/mutation of the target site. HGT has an essential role in microorganism evolution and spread of resistance genes against different antimicrobial agents and is a main cause of genome expansion. Mobile genetic elements in *P. aeruginosa* transmit a wealth of genes that have been implied in a range of attributes including virulence formation (Louwen et al. 2014), xenobiotics degeneration, and antimicrobial resistance (van Belkum et al. 2015).

CRISPR systems were initially known for their role in phage defense through inhibition of lysogenic conversion that is a principal mechanism of HGT. Later on, CRISPR/Cas systems were recognized for targeting other mobile genetic elements including plasmids or transformed DNA or integrative conjugative elements (Silveira et al. 2020; van Belkum et al. 2015; Wheatley and MacLean 2021). A previous study has indicated that strains of *P. aeruginosa* harboring CRISPR systems have considerably smaller genome sizes along with lowered mobile sulphonamide resistance genes (Shehreen et al. 2019).

Recent studies have indicated that strains carrying CRISPR/Cas systems are characterized by smaller genome sizes and elevated GC content indicating that they hamper the transfer/gain of mobile genetic elements. The majority of strains harboring CRISPR systems have spacers targeting ICE and the conserved machinery for conjugative transfer of ICE and plasmids. Moreover, genomes harboring CRISPR/Cas systems have lowered the abundance of ICE and prophages. Remarkably, spacers of CRISPR systems delineate phages and ICE incorporated into the genome of strains lacking these systems (Silveira et al. 2020; van Belkum et al. 2015; Wheatley and MacLean 2021).

In summary, this study provides a novel/innovative method for the detection of CRISPR/Cas-positive strains of *P. aeruginosa* via PCR and in a single reaction tube. We hope that this strategy would help to easily identify CRISPR/Cas-positive strains of *P. aeruginosa*. We believe that such convenience in identification will contribute to a better understanding of the correlational studies between the existence of CRISPR systems in *P. aeruginosa* and other characters of such pathogens, e.g., virulence, antibiotic resistance, and adaptation to environmental stress.

## Supplementary Information

Below is the link to the electronic supplementary material.

Supplementary file1 (PDF 8911 KB)

## Data Availability

All data generated and analyzed during this study are included in this article and its supplementary information files.
