# Supplementary material for: Novel PCR detection of CRISPR/Cas systems in Pseudomonas aeruginosa and its correlation with antibiotic resistance
Source: Appl Microbiol Biotechnol. 2022 Sep 30;106(21):7223–34. doi: 10.1007/s00253-022-12144-1 (PMC9592639; doi:10.1007/s00253-022-12144-1)

Applied Microbiology and Biotechnology

Novel PCR-detection of CRISPR-Cas systems in *Pseudomonas aeruginosa* and its correlation with antibiotic resistance

Mai Soliman, Heba Shehta Said, Mohammed El-Mowafy, and Rasha Barwa

Department of Microbiology and Immunology, Faculty of Pharmacy,

Mansoura University, Mansoura 35516, Egypt.

Corresponding authors:

Heba Shehta Said, Ph.D.

Department of Microbiology and Immunology, Faculty of Pharmacy, Mansoura University, Mansoura 35516, Egypt. E-mail: hebashehta@mans.edu.eg or hebashehta@yahoo.com

Mohammed El-Mowafy, Ph.D.

Department of Microbiology and Immunology, Faculty of Pharmacy, Mansoura University, Mansoura 35516, Egypt. E-mail: seven@mans.edu.eg

Rasha Barwa, Ph.D.

Department of Microbiology and Immunology, Faculty of Pharmacy, Mansoura University, Mansoura 35516, Egypt. E-mail: rasha@mans.edu.eg or rasha2000@gmail.com

Table S1: General information about *P. aeruginosa* genomes and CRISPR-Cas genes retrieved from NCBI.

CRISPR-Cas subtype	Gene	Accession number	Isolation niche	Source	Geographical location of sample collection	Sex
I-C	*cas8*	ALP58421	Clinical	Skin wound	Unknown	---
		QEK45103	Clinical	Urine	Sweden	---
		QCP79067	Clinical	Feces	China	---
		TGN53427	Clinical	Unknown	Brazil	---
		TEX35268	Environmental	Sandy soil included hydrocarbon	Canada	---
		RYI94172	Clinical	Secretion swab	Brazil	---
		RPW43362	Environmental	Sea water (open ocean)	Japan	---
		RRI32906	Clinical	Bronchoalveolar lavage	United kingdom	---
		PYB39273	Clinical	Blood	Brazil	---
		PKG10847	Environmental	River	Brazil	---
		PCB89278	Environmental	Soil	USA	---
		OZO32239	Environmental	Open ocean	Pacific Ocean	---
		TQR62362	Clinical	Sputum	USA	---
		RCA28664	Clinical	Military dog fecal sample	Germany	---
		OWI94047	Clinical	Infection control	USA	---
		WP_134612090	Clinical	---	Spain	---
		WP_033993299	Bovine	Milk	Estonia	---
		WP_033985464	Clinical	Wound	USA	---
		WP_023119728	Clinical	Skin wound	USA	---
		WP_023104936	Clinical	Conjunctiva	USA	---
		WP_025981579	Clinical	Bronchoalveolar lavage	United kingdom	---
		WP_031631553	Clinical	Urine	France	---
		QIU80118	---	---	---	---
		PBN23468	Clinical	Infected lung	Canada	---
		KSM52103	Clinical	Unknown	USA	---
	cas5	AOX29156	Clinical	Urine	Brazil	---
		QCP79068	Clinical	Feces	China	---
		TGN53428	Clinical	---	Brazil	---
		TEX35267	Environmental	Sandy soil included hydrocarbon	Canada	---
		RYI94173	Clinical	Secretion swab	Brazil	---
		RTV01284	Clinical	Groin	USA	---
		RRI32905	Clinical	Cystic fibrosis	United kingdom	---
		RPY98922	Environmental	Soil	Switzerland	---
		PYB39272	Clinical	Blood	Brazil	---
		PKG10848	Environmental	River	Brazil	---
		PCB89277	Environmental	Soil	USA	---
		OZO32240	Environmental	Open ocean	Pacific Ocean	---
		QIU80117	---	---	---	---
		TQR62361	Clinical	Sputum	USA	---
		WP_089035047	Clinical	Ear secretion -dog	Estonia	---
		WP_077144399	Clinical	Airway secretions	USA	Female
		WP_023104937	Clinical	Bronchoalveolar lavage	USA	---
		WP_031631556	Clinical	Urine	France	---
		KAA5578455	Clinical	Sputamentum	China	---
		OWI94046	Clinical	Infection control	USA	---
		RCA28663	Clinical	Military dog fecal sample	Germany	---
		RIY90655	---	---	Hong Kong	---
		QKR37308	Clinical	Lung	USA	---
		ALP58420	Clinical	Skin wound of burn patient	---	---
		KSC27646	Clinical	Hospital	USA	---
		OTF49166	Clinical	Urine	Brazil	---
	cas7	ARU35259	Clinical	Catheter tip	Brazil	---
		QIU80119	---	---	---	---
		QEK45102	Clinical	Urine	Sweden	---
		QCP79066	Clinical	Human feces	China	---
		TEO46103	Clinical	Nasopharynx	Canada	Female
		RYM12515	Clinical	Urine	Brazil	---
		RYI94171	Clinical	Secretion swab	Brazil	---
		RTX37063	Clinical	Wound	USA	---
		RRI32907	Clinical	Cystic fibrosis	United kingdom	---
		RQJ47602	Clinical	Shin ulcer	United kingdom	---
		PYB39274	Clinical	Blood	Brazil	---
		PKG15190	Environmental	River	Brazil	---
		OWI94048	Clinical	Infection control	USA	---
		RCA28665	Clinical	Military dog fecal sample	Germany	---
		RIY90653	---	---	Hong Kong	---
		WP_023104935	Clinical	Conjunctiva	USA	---
		WP_077144400	Clinical	Airway secretions	USA	Female
		TQR62363	Clinical	Sputum	USA	---
		KAA5578453	Clinical	Sputamentum	China	---
		ALP58422	Clinical	Skin wound of burn patient	---	---
		KSC27644	Clinical	Hospital	USA	---

		OTF49167	Clinical	Urine	Brazil	---
		OZO32238	Environmental	Open ocean	Pacific Ocean	---
		PAT16604	Environmental	Blueberry-like berries	USA	---
		PBM48442	---	Dental unit waterline	Canada	---
I-E	*cas5*	TRO94856	---	Cell culture	USA	---
		TRM22435	---	Cell culture	USA	---
		TGP00796	Clinical	Wound swab	Sudan	---
		TER84128	Clinical	Sputum	Canada	Female
		RWY44024	Clinical	Sputum	Brazil	---
		RTX61657	Clinical	Respiratory	USA	---
		RRJ11364	Clinical	Cystic fibrosis patient	United kingdom	---
		KAA5643264	Clinical	Sputamentum	China	---
		QDR03599	Clinical	Sputum	China	---
		QDD51228	---	Water bottle, Animal room	Unknown	---
		QCJ31864	---	Water bottle, Animal room	Unknown	---
		RTC47438	---	---	---	---
		QKR09497	Clinical	Lung	USA	---
		QJW76813	Clinical	Sepsis/ hemoculture	Mexico	---
		QIB87118	Clinical	Pus	India	---
		QHF90445	---	Tobacco-single cell	China	---
		QGJ34878	Clinical	Urine	USA	---
		QFZ60303	Clinical	Sputum	Thailand	---
		TRM04454	---	Cell culture	USA	---
		TQO72222	Environmental	Sea water	Mexico	---
		RQI92595	Clinical	Heel ulcer	United kingdom	---
		RNL96164	Clinical	Urine	Hungary	---
		PUA11408	Clinical	Blood	Brazil	---
		AVN46668	---	---	---	---
		PCC06504	Environmental	Sink faucet	USA	---
	cas7	AMA38574	Clinical	Patient nose	France	---
		QJW76812	Clinical	Sepsis/ hemoculture	Mexico	---
		QIB87117	Clinical	Pus	India	---
		QHF90446	---	Tobacco-single cell	China	---
		QGJ34879	Clinical	Urine	USA	---
		QFZ60304	Clinical	Sputum	Thailand	---
		TRO94855	---	Cell culture	USA	---
		TRM22436	---	Cell culture	USA	---
		TRM04453	---	Cell culture	USA	---
		TQO72221	Environmental	Sea water	Mexico	---
		TGP00795	Clinical	Wound swab	Sudan	---
		TER84129	Clinical	Sputum	Canada	Female
		ARN36484	Environmental	Crude oil residual water	Colombia	---
		OTI64064	Clinical	Blood	Italy	---
		OWI71360	Clinical	Blood	USA	---
		PCN23865	Clinical	Wound swab	Ghana	Male
		PLB07980	Clinical	Urinary catheter	USA	---
		PNP69849	Clinical	Urinary catheter	USA	Female
		AVE34530	Clinical	Urine	USA	---
		PTV67798	Clinical	---	USA	---
		AWR43411	Clinical	Sputum	Sweden	---
		RIY98354	---	---	Hong Kong	---
		AYN81759	---	---	China	---
		RTA74410	---	---	---	---
		KAA5643265	Clinical	Sputamentum	China	---
	cas11	TGP00794	Clinical	Wound swab	Sudan	---
		TER84130	Clinical	Sputum	Canada	Female
		RWY44022	Clinical	Sputum	Brazil	---
		RTX61655	Clinical	Respiratory	USA	---
		RRJ11366	Clinical	Cystic fibrosis patient	United kingdom	---
		QDD51226	---	Water bottle, Animal room	Unknown	---
		QCJ31866	---	Water bottle, Animal room	Unknown	---
		RTC47440	---	---	---	---
		RNL96162	Clinical	Urine	Hungary	---
		PUA11410	Clinical	Blood	Brazil	---
		AVN46666	---	---	---	---
		AUA96383	Clinical	Abscess	USA	---
		PCC06506	Environmental	Sink faucet	USA	---
		PXA59808	Environmental	Sea water	United kingdom	---
		AWR43412	Clinical	Sputum	Sweden	---
		AYN81758	---	---	China	---
		ASC98811	Clinical	Bronchial washing	Mexico	---
		PTV91357	---	Coyote-single bacteria	USA	---
		AVE34531	Clinical	Urine	USA	---
		PLB07981	Clinical	Urinary catheter	USA	---
		PCN23864	Clinical	Wound swab	Ghana	Male
		OWI74403	Clinical	Blood	USA	---
		QJW76811	Clinical	Sepsis/ hemoculture	Mexico	---
		QIB87116	Clinical	Pus	India	---
		TKW45018	Clinical	Wound	Malaysia	---
		KAB0789151	---	---	USA	---
		KAA8774467	Environmental	Water	South Africa	---

I-F	*cas5*	KAA5656880	Clinical	Sputamentum	China	---
		QDR17086	Clinical	Bile	China	---
		TYT09336	Clinical	Tracheal secretions	Norway: Oslo	---
		TWW31629	Clinical	Cornea	USA	---
		TSB12740	Clinical	Peritoneal fluid	Peru	---
		TRO98215	---	Cell culture	USA	---
		TRO86129	---	Cell culture	USA	---
		TRM22854	---	Cell culture	USA	---
		TRL97048	---	Cell culture	USA	---
		PWU34855	Environmental	River sediment	Germany	---
		AYF69994	Clinical	Blood	India	---
		TJY50170	Clinical	---	Turkey	---
		TEX70314	Clinical	Sputum	Canada	Female
		RWX95209	Clinical	Bone fragment	Brazil	---
		RTX24272	Clinical	Urine	USA	---
		RTB82905	Unknown	Unknown	Unknown	---
		RRJ57268	Clinical	Cystic fibrosis patient	United kingdom	---
		AYZ85115	Clinical	UCC isolate	Unknown	---
		QEF90254	Environmental	Crude oil	China	---
		TQR65330	Clinical	Sputum	USA	---
		QBL19722	Clinical	BAL	France	---
		RKG24931	Clinical	Suppuration disease	Cote d'Ivoire: Abidjan	---
	cas7	KAB0789152	Unknown	Unknown	USA	---
		KAA5656879	Clinical	Sputamentum	China	---
		QDR08646	Clinical	Sputum	China	---
		TRL54026	---	Cell culture	USA	---
		QDD52552	Environmental	Water bottle, Animal room	Unknown	---
		QCJ30551	Environmental	Water bottle, Animal room	Unknown	---
		TGB16781	Clinical	Wound	Myanmar	Male
		TEN69357	Clinical	Sputum	Canada	Female
		RZN96665	Clinical	Urine	Spain	---
		RWY48827	Clinical	Tissue fragment	Brazil	---
		RTT63656	Clinical	Urine	USA	---
		RRJ04415	Clinical	Cystic fibrosis patient	United kingdom	---
		RPM66457	Clinical	Leg wound	United kingdom	---
		RKG24932	Clinical	Suppuration disease	Cote d'Ivoire: Abidjan	---
		RCM99250	Clinical	Lung	Australia	---
		RCA23072	Clinical	Military dog fecal sample	Germany	---
		AWZ92308	Clinical	---	---	---
		AWT29008	Clinical	Tracheal secretions	Norway: Oslo	---
		AWS90229	Clinical	---	---	---
		PUV68294	Clinical	Blood	Brazil	---
		AVR67830	Environmental	Chilli rhizosphere	India	---
		PQL96533	Clinical	Blood	Australia	---
		POO65035	Environmental	Water	India	---
		PNN38791	Clinical	Endotracheal aspirate	USA	Female
		PHP80844	Clinical	---	USA	---
	cas8	TKW45019	Clinical	Wound	Malaysia	---
		KAB0772843	Unknown	Unknown	USA	---
		KAA5656881	Clinical	Sputamentum	China	---
		TSB12739	Clinical	Peritoneal fluid	Peru	---
		TRO98216	---	Cell culture	USA	---
		TRM22855	---	Cell culture	USA	---
		TRL97047	---	Cell culture	USA	---
		QDD52550	Environmental	Water bottle, Animal room	Unknown	---
		AYF69995	Clinical	Blood	India	---
		QCJ30553	Environmental	Water bottle, Animal room	Unknown	---
		TJY50171	Clinical	---	Turkey	---
		TGB16779	Clinical	Wound	Myanmar	Male
		TEX70315	Clinical	Sputum	Canada	Female
		RZN96663	Clinical	Urine	Spain	---
		RWY53499	Clinical	Urine	Brazil	---
		RTS97243	Clinical	---	USA	---
		RTC05193	Clinical	Unknown	Unknown	---
		AZM86033	Clinical	Urine	Belgium	---
		RRJ46631	Clinical	Cystic fibrosis patient	United kingdom	---
		AWT29010	Clinical	Tracheal secretions	Norway: Oslo	---
		AYZ85114	Clinical	UCC isolate	Unknown	---
		QEF90255	Environmental	Crude oil	China	
		TQR65331	Clinical	Sputum	USA	---
		AYL32227	Clinical	Unknown	China	---
		QBL24424	Clinical	BAL	France	---

Table S2: Characterization of all *P. aeruginosa* clinical isolates based on collection data and its antimicrobial susceptibility and biofilm formation patterns.

	Isolate code	Clinical source	Date of isolation	Gender	CRISPR-Cas subtype	Antimicrobial class									Biofilm category
						β-lactams						Aminoglycosides	Quinolones		
						Penicillins	β-lactam comination agents	Cephalosporins		Carbapenems					
						PRL	TPZ	CAZ	FEP	IPM	MEM	AK	CIP	LEV	
CRISPR-Cas positive isolates	Cc1	Wound	06/08/20	Female	I-C	S	S	R	R	S	S	S	S	S	MP
	Cc2	Pus	09/08/20	Male	I-C	R	R	R	R	R	R	R	R	R	NP
	Cc3	BAL	25/8/2020	Male	I-C	R	R	R	R	R	R	R	R	R	SP
	Cc4	Urine	09/09/20	Male	I-C	S	S	R	S	S	S	S	S	S	MP
	Cc5	Urinary catheter	20/9/2020	Male	I-C	R	R	R	R	R	R	R	S	S	MP
	Cc6	Urinary catheter	20/9/2020	Female	I-C	S	S	R	R	S	S	S	S	S	WP
	Cc7	Urinary catheter	24/9/2020	Female	I-C	S	S	R	R	S	S	S	S	S	SP
	Cc8	Urine	26/10/2020	Female	I-C	S	S	R	R	S	S	S	S	S	WP
	Cc9	Corneal swab	15/11/2020	Female	I-C	S	S	R	R	S	S	S	S	S	NP
	Cc10	Urine	24/12/2020	Female	I-C	S	S	R	R	S	S	S	S	S	WP
	Ec1	ETA	25/6/2020	Male	I-E	R	R	R	R	R	R	R	R	R	WP
	Ec2	Blood	25/6/2020	Male	I-E	S	S	R	R	S	S	S	S	S	SP
	Ec3	Blood	26/6/2020	Female	I-E	S	S	R	R	S	S	S	S	S	MP
	Ec4	ETA	27/6/2020	Female	I-E	S	S	R	R	S	S	S	S	S	SP
	Ec5	Wound	05/07/20	Male	I-E	S	S	R	R	S	S	S	S	S	SP
	Ec6	Urine	09/07/20	Male	I-E	S	S	R	R	S	S	S	S	S	MP
	Ec7	Urine	04/08/20	Female	I-E	R	R	R	R	R	R	R	R	R	MP
	Ec8	Vaginal swab	18/11/2020	Female	I-E	S	S	R	R	S	S	S	S	S	MP
	Ec9	Urine	22/12/2020	Male	I-E	S	S	R	R	S	S	S	S	S	WP
	Fc1	Urinary catheter	06/08/20	Male	I-F1	S	S	R	R	S	S	S	S	S	MP
	Fc2	Urine	06/08/20	Female	I-F1	S	S	R	R	S	S	S	S	S	MP
	Fc3	Urinary catheter	07/08/20	Female	I-F1	S	S	R	R	S	S	S	S	S	WP
	Fc4	Wound	08/08/20	Female	I-F1	R	R	R	R	R	R	S	R	R	WP
	Fc5	Urine	11/08/20	Male	I-F1	S	S	R	R	S	S	S	S	S	MP
	Fc6	Pus	22/8/2020	Female	I-F1	R	R	R	R	R	R	S	R	R	MP
	Fc7	Urine	22/8/2020	Male	I-F1	S	S	R	R	S	S	S	S	S	WP
	Fc8	Urine	05/09/20	Female	I-F1	S	S	R	R	S	S	S	S	S	MP
	Fc9	Urinary catheter	11/09/20	Male	I-F1	S	S	R	R	S	S	S	S	S	MP
	Fc10	Urinary catheter	20/9/2020	Female	I-F1	S	S	R	R	S	S	S	S	S	NP
	Fc11	Sputum	20/9/2020	Male	I-F1	S	S	R	R	S	S	S	S	S	SP
	Fc12	Urine	18/11/2020	Male	I-F1	R	R	R	R	R	R	R	S	S	NP
	Fc13	Urine	19/12/2020	Male	I-F1	S	S	R	R	S	S	S	R	R	NP
	Nc3	Urine	23/6/2020	Female	-	R	S	S	S	S	S	S	S	S	WP
	Nc5	Urine	25/6/2020	Female	-	R	R	R	R	R	R	R	R	R	NP
	Nc10	Urine	05/07/20	Male	-	R	S	R	S	S	S	S	S	S	NP
	Nc12	Urine	05/07/20	Female	-	S	S	R	S	S	S	S	S	S	WP
	Nc13	Urine	11/07/20	Female	-	S	S	R	S	S	S	S	S	S	SP
	Nc16	Urine	16/7/2020	Female	-	S	S	R	R	S	S	S	S	S	NP
	Nc22	Urine	26/7/2020	Male	-	S	S	R	R	S	S	S	S	S	WP

CRISPR-Cas negative isolates	Nc26	Urine	11/08/20	Female	-	R	S	S	S	S	S	S	R	R	MP
	Nc27	Urine	23/8/2020	Male	-	R	S	S	S	S	S	S	S	R	NP
	Nc29	Urine	25/8/2020	Male	-	R	S	S	S	S	S	S	S	S	NP
	Nc30	Urine	27/8/2020	Female	-	R	S	S	S	S	S	S	S	S	NP
	Nc32	Urine	27/8/2020	Female	-	S	S	R	S	R	R	S	S	S	NP
	Nc33	Urinary catheter	30/8/2020	Female	-	S	S	R	R	S	S	S	S	S	WP
	Nc34	Urinary catheter	30/8/2020	Male	-	R	R	R	R	R	R	R	R	R	SP
	Nc36	Urinary catheter	05/09/20	Female	-	R	S	R	S	S	S	S	S	S	MP
	Nc38	Urinary catheter	08/09/20	Male	-	S	S	R	R	S	S	S	R	R	WP
	Nc39	Urinary catheter	08/09/20	Male	-	R	R	R	R	R	S	R	R	R	WP
	Nc40	Urinary catheter	13/9/2020	Female	-	R	S	R	R	R	R	R	R	R	NP
	Nc41	Urinary catheter	15/9/2020	Male	-	R	S	R	S	R	R	S	S	S	NP
	Nc52	Wound	12/10/20	Male	-	R	S	R	S	S	S	S	S	S	MP
	Nc57	Wound	26/10/2020	Male	-	R	S	R	R	S	S	S	S	S	WP
	Nc59	Wound	02/11/20	Male	-	R	R	R	R	R	R	R	R	R	NP
	Nc42	ETA	03/09/20	Female	-	R	S	R	S	S	S	S	S	S	WP
	Nc43	ETA	08/09/20	Female	-	R	S	R	S	S	S	S	S	S	WP
	Nc45	BAL	09/09/20	Female	-	R	S	R	S	S	S	S	S	S	NP
	Nc49	Sputum	21/9/2020	Female	-	R	S	R	S	S	S	S	S	S	SP
	Nc66	Pus	12/12/20	Male	-	R	R	R	R	R	R	R	R	R	NP
	Nc68	Pus	19/12/2020	Male	-	R	S	R	S	S	S	S	S	S	MP
	Nc75	Blood	22/12/2020	Female	-	R	S	R	S	S	S	S	S	S	WP
	Nc79	Blood	24/12/2020	Female	-	R	S	R	S	S	S	S	S	S	WP
	Nc90	Vaginal swab	26/12/2020	Female	-	R	R	R	R	R	R	R	R	R	WP
	Nc84	Corneal swab	26/12/2020	Male	-	R	S	R	S	S	S	S	S	S	MP
ates	Nc1	Urine	26/6/2020	Male											
	Nc2	Urine	26/6/2020	Female											
	Nc4	Urine	06/08/20	Female											
	Nc6	Urine	11/08/20	Male											
	Nc7	Urine	15/8/2020	Female											
	Nc8	Urine	15/8/2020	Male											
	Nc9	Urine	03/09/20	Female											
	Nc11	Urine	17/9/2020	Male											
	Nc14	Urine	17/9/2020	Female											
	Nc15	Urine	03/10/20	Male											
	Nc17	Urine	03/10/20	Male											
	Nc18	Urine	15/10/2020	Male											
	Nc19	Urine	15/10/2020	Female											
	Nc20	Urine	20/10/2020	Male											
	Nc21	Urine	20/10/2020	Male											
	Nc23	Urine	26/10/2020	Male											
	Nc24	Urine	30/10/2020	Female											
	Nc25	Urine	15/11/2020	Female											
	Nc28	Urine	17/11/2020	Male											
	Nc31	Urine	20/11/2020	Male											
	Nc35	Urinary catheter	15/12/2020	Female											
	Nc37	Urinary catheter	06/08/20	Male											
	Nc44	BAL	03/09/20	Male											

Other CRISPR-Cas negative isola	Nc46	BAL	08/09/20	Female
	Nc47	BAL	08/09/20	Male
	Nc48	BAL	17/11/2020	Female
	Nc50	Sputum	26/6/2020	Male
	Nc51	Wound	05/07/20	Male
	Nc53	Wound	05/07/20	Female
	Nc54	Wound	06/08/20	Male
	Nc55	Wound	11/08/20	Male
	Nc56	Wound	11/08/20	Female
	Nc58	Wound	15/8/2020	Male
	Nc60	Burn	05/08/20	Male
	Nc61	Burn	08/09/20	Female
	Nc62	Burn	08/09/20	Male
	Nc63	Burn	15/10/2020	Female
	Nc64	Burn	20/10/2020	Male
	Nc65	Burn	14/11/2020	Female
	Nc67	Pus	02/12/20	Male
	Nc69	Pus	26/12/2020	Female
	Nc70	Blood	25/6/2020	Male
	Nc71	Blood	16/7/2020	Female
	Nc72	Blood	25/7/2020	Male
	Nc73	Blood	08/08/20	Female
	Nc74	Blood	11/08/20	Male
	Nc76	Blood	23/8/2020	Female
	Nc77	Blood	05/09/20	Male
	Nc78	Blood	17/9/2020	Male
	Nc80	Blood	17/9/2020	Male
	Nc81	Blood	24/9/2020	Female
	Nc82	Blood	18/11/2020	Female
	Nc83	Contact lens	20/11/2020	Female
	Nc85	Eye	25/6/2020	Female
	Nc86	Ear	08/08/20	Male
	Nc87	Vaginal swab	11/08/20	Female
	Nc88	Vaginal swab	15/8/2020	Female
	Nc89	Vaginal swab	15/12/2020	Female

C: CRISPR-Cas subtype I-C**, E:** CRISPR-Cas subtype I-E**, F:** CRISPR-Cas subtype I-F1
c: clinical isolate**, Nc:** CRISPR-Cas negative clinical isolate
BAL: Brochoalveolar Lavage, **ETA:** Endotracheal Aspirate
S: sensitive**, R:** resistant, **PRL:** piperacillin, **TPZ:** piperacillin-tazobactam, **CAZ :** ceftazidime, **FEP:** cefepime, **IPM:** imipenem, **MEM:** meropenem, **AK:** amikacin, **CIP:** ciprofloxacin, **LEV:** levofloxacin
SP: strong biofilm producer, **MP**: moderate biofilm producer, **WP**: weak biofilm producer, **NP:** non biofilm producer

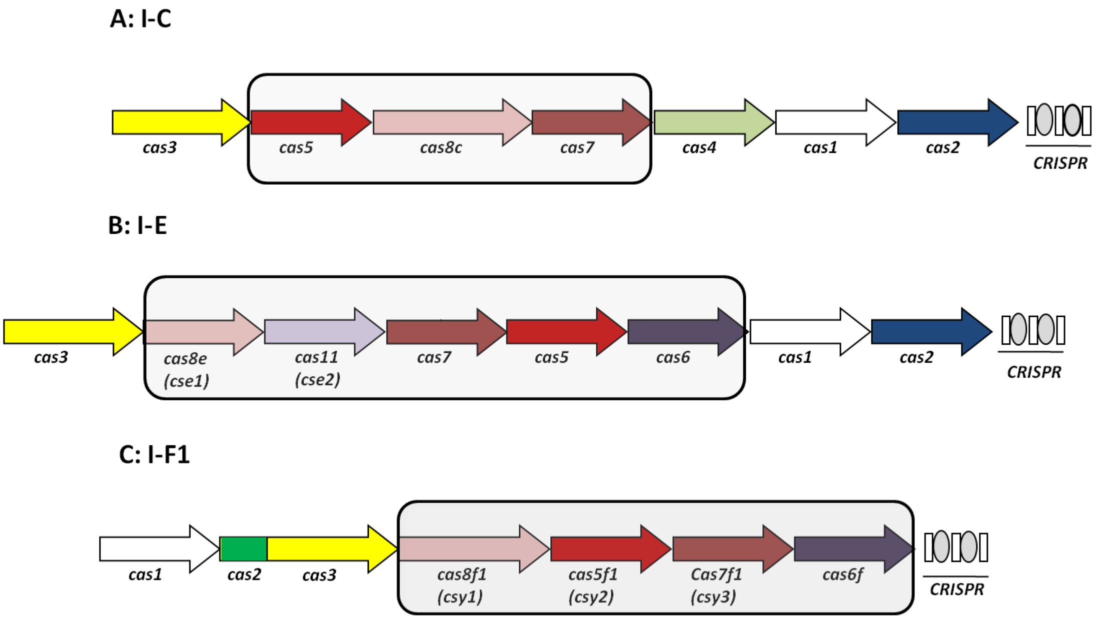

Fig. S1: Schematic representation of CRISPR-Cas loci of known subtypes in *P. aeruginosa*: I-C (A), I-E (B) and I-F1 (C). Genes included in the grey rectangle represents the effector proteins.

Fig. S2: Analysis of multiplesequence alignments of 25 full gene sequences of *P. aeruginosa* strains in the CRISPR-Cas cassettes of different subtypes showing the target conserved regions for primers design.

A) Subtype I-C_*cas5* gene B) Subtype I-C_*cas7* gene C) Subtype I-C_*cas8* gene

D) Subtype I-E_*cas5* gene E) Subtype I-E_*cas7* gene F) Subtype I-E_*cas11* gene

G) Subtype I-F1_*cas5* gene H) Subtype I-F1_*cas7* gene I) Subtype I-F1_*cas8* gene

The ID of each full gene sequence was named by the subtype followed by its accession number in the GenBank.

A

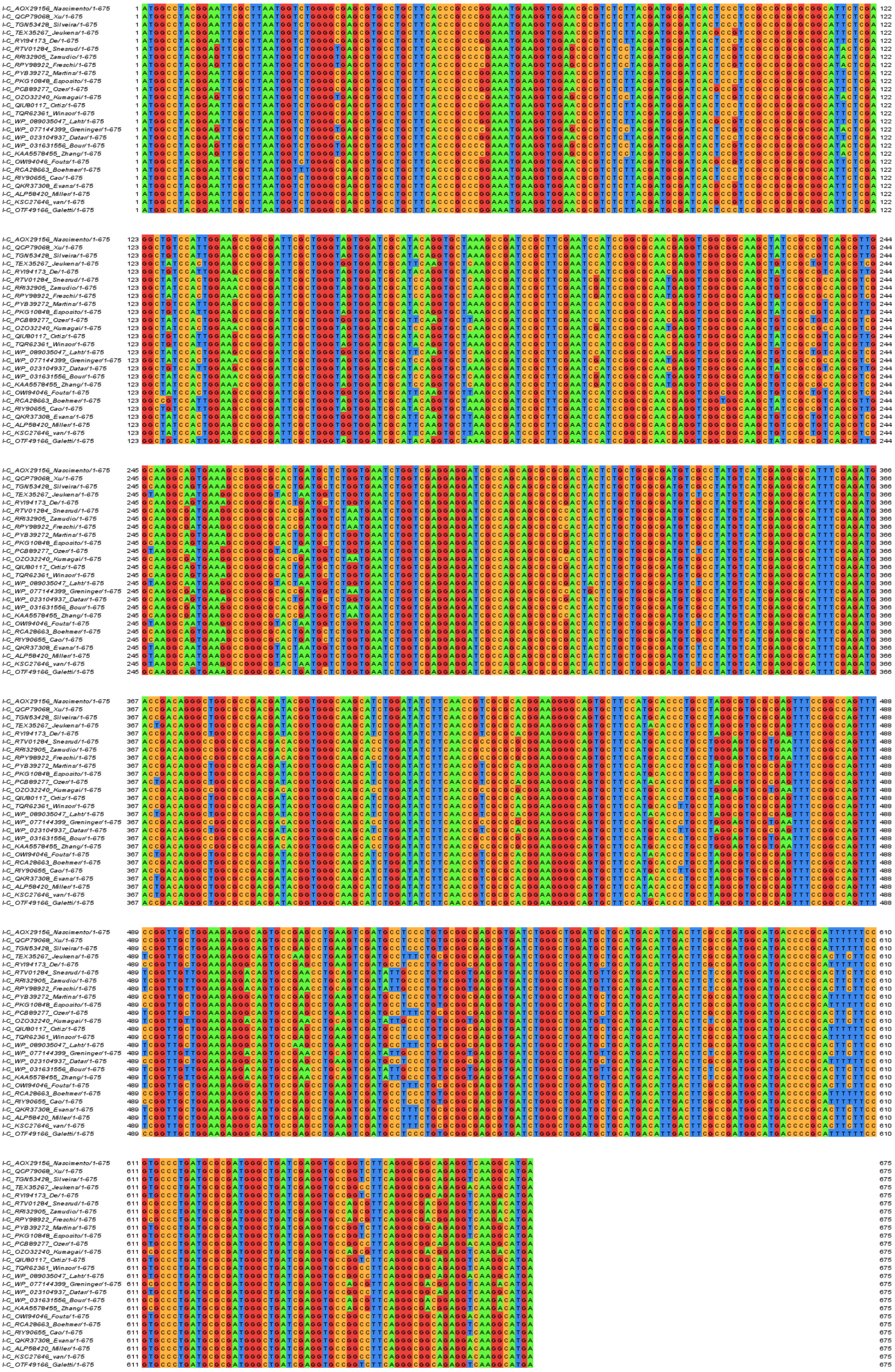

B

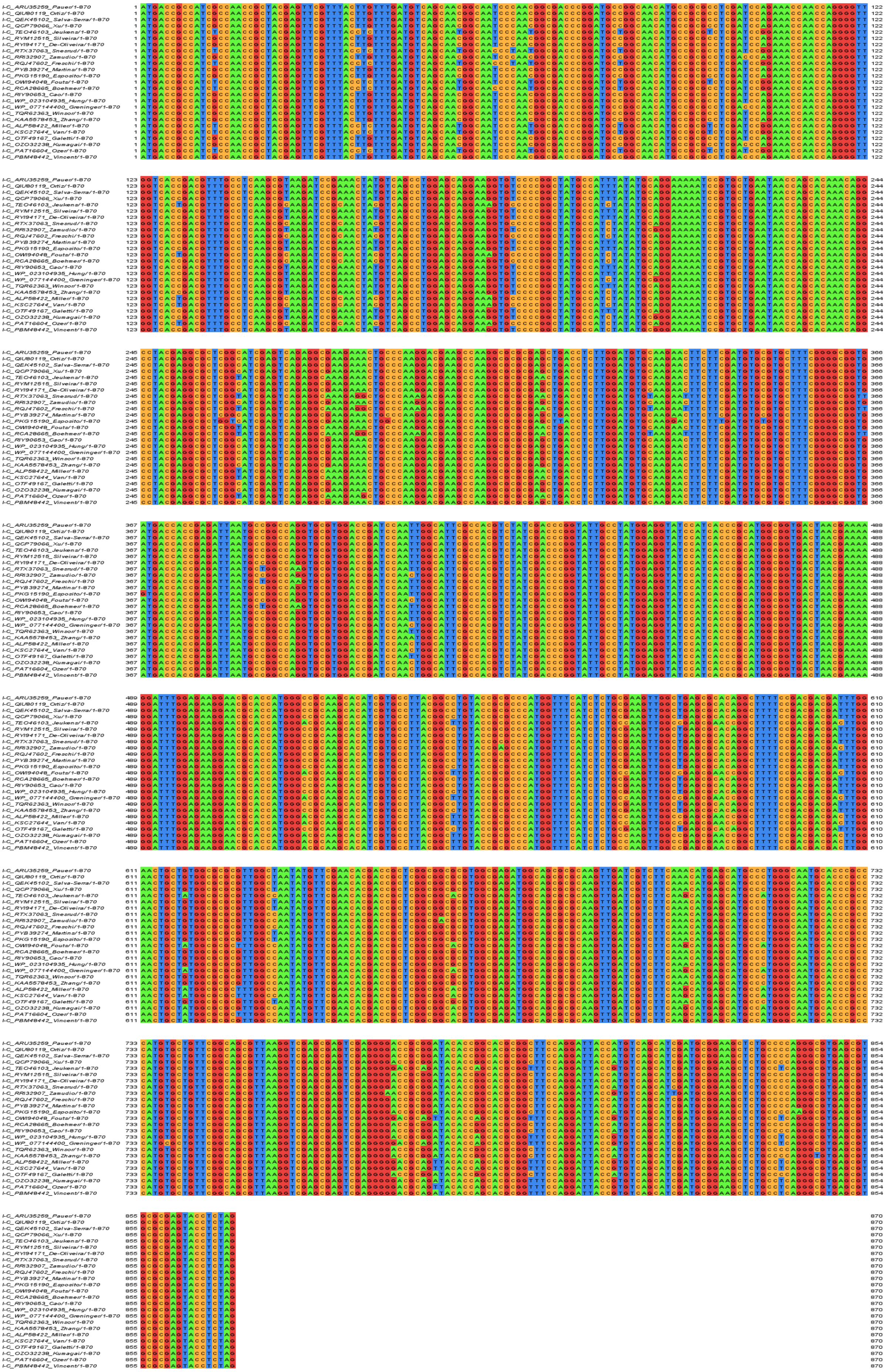
I-C_ARU35259_Pauer/1-870
I-C_QIU80119_Ortiz/1-870
I-C_QEK45102_Salva-Serra/1-870
I-C_QCP79066_Xu/1-870
I-C_TEO46103_Jeukens/1-870
I-C_RYM12515_Silveira/1-870
I-C_RYI94171_De-Oliveira/1-870
I-C_RTX37063_Snesrud/1-870
I-C_RRI32907_Zamudio/1-870
I-C_RQJ47602_Freschi/1-870
I-C_PYB39274_Martins/1-870
I-C_PKG15190_Esposito/1-870
I-C_OWI94048_Fouts/1-870
I-C_RCA28665_Boehmer/1-870
I-C_RIY90653_Cao/1-870
I-C_WP_023104935_Hung/1-870
I-C_WP_077144400_Greninger/1-870
I-C_TQR62363_Winsor/1-870
I-C_KAA5578453_Zhang/1-870
I-C_ALP58422_Miller/1-870
I-C_KSC27644_Van/1-870
I-C_OTF49167_Galetti/1-870
I-C_OZO32238_Kumagai/1-870
I-C_PAT16604_Ozer/1-870
I-C_PBM48442_Vincent/1-870

C

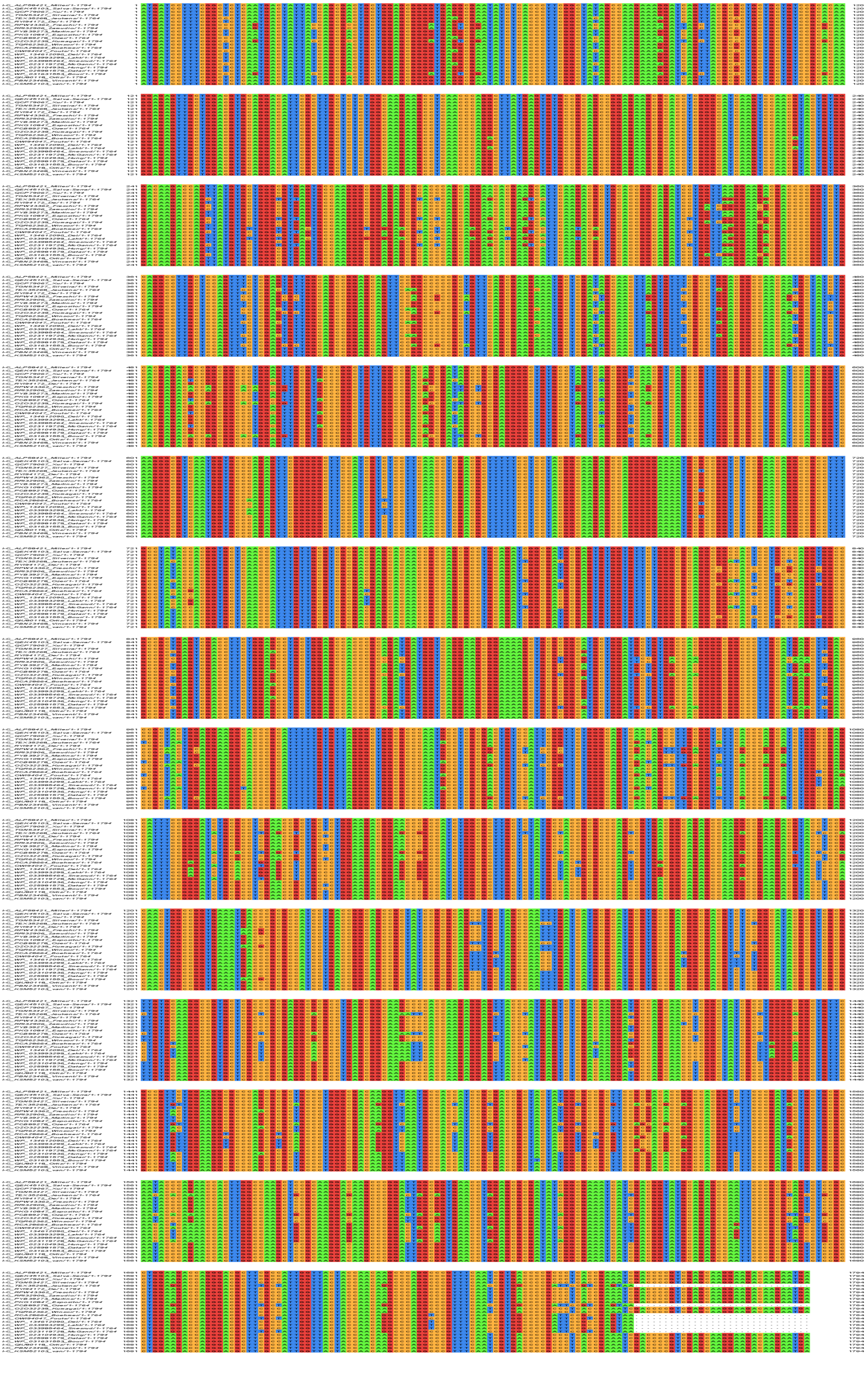

D

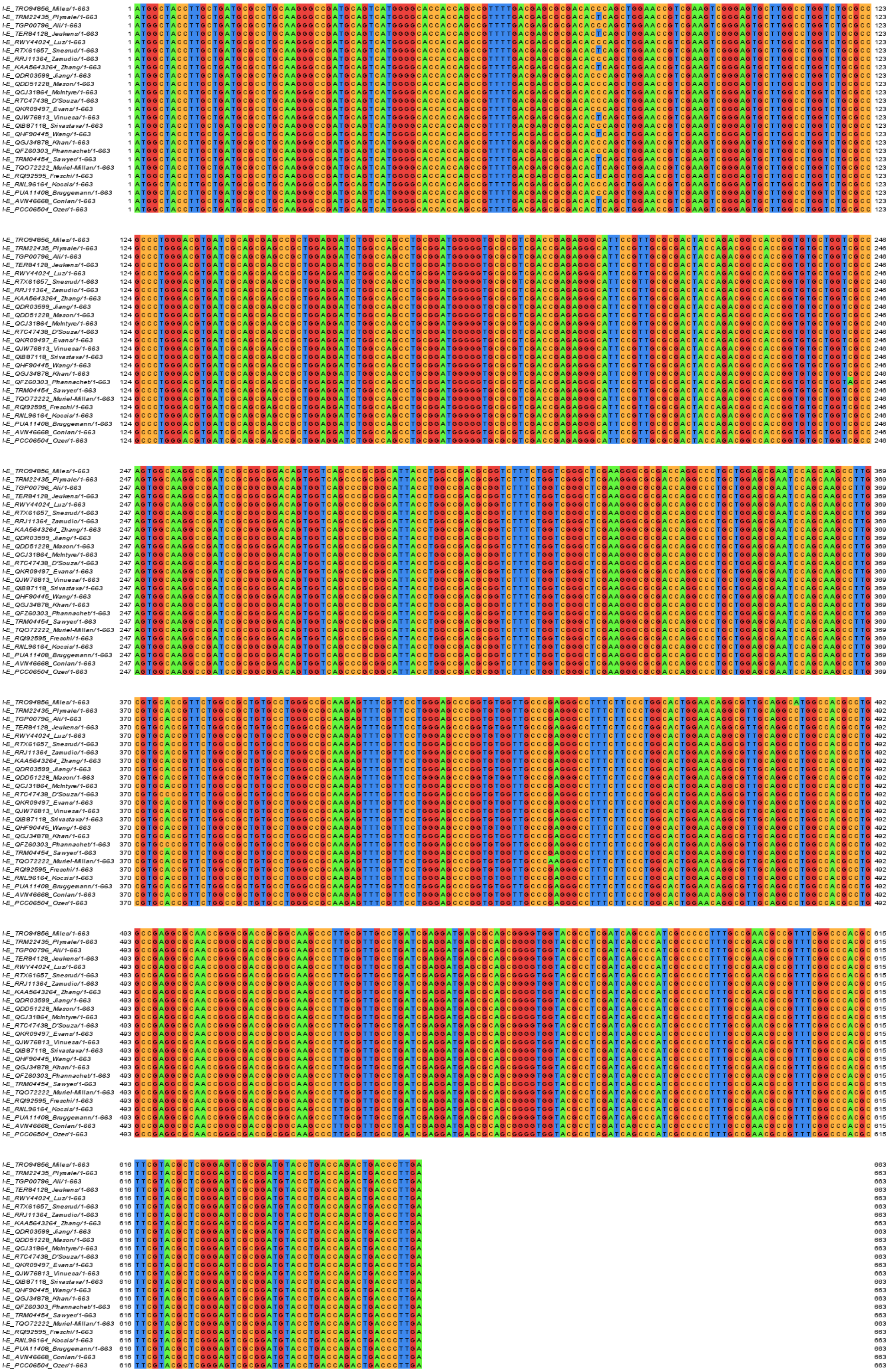

E

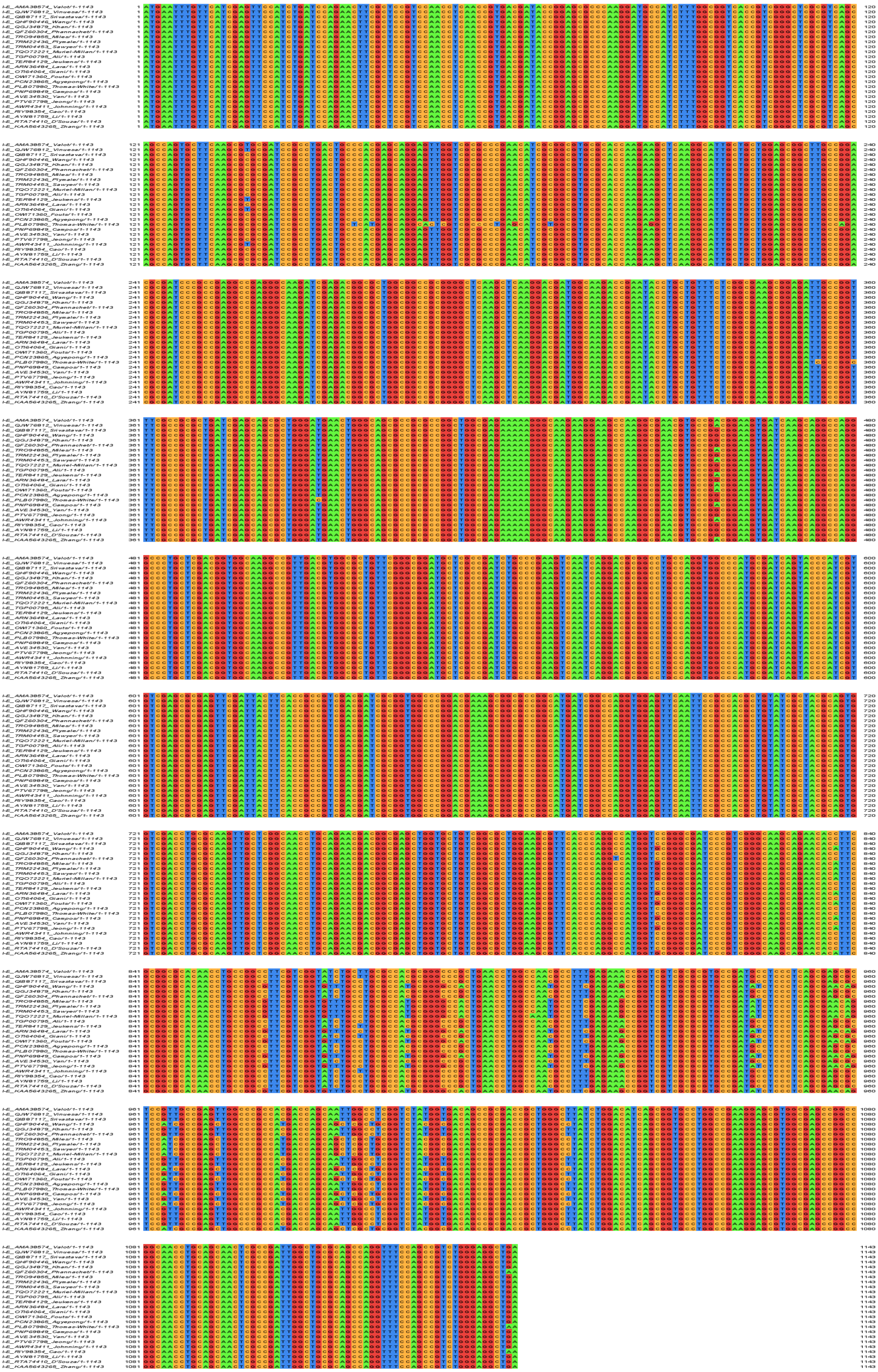

F

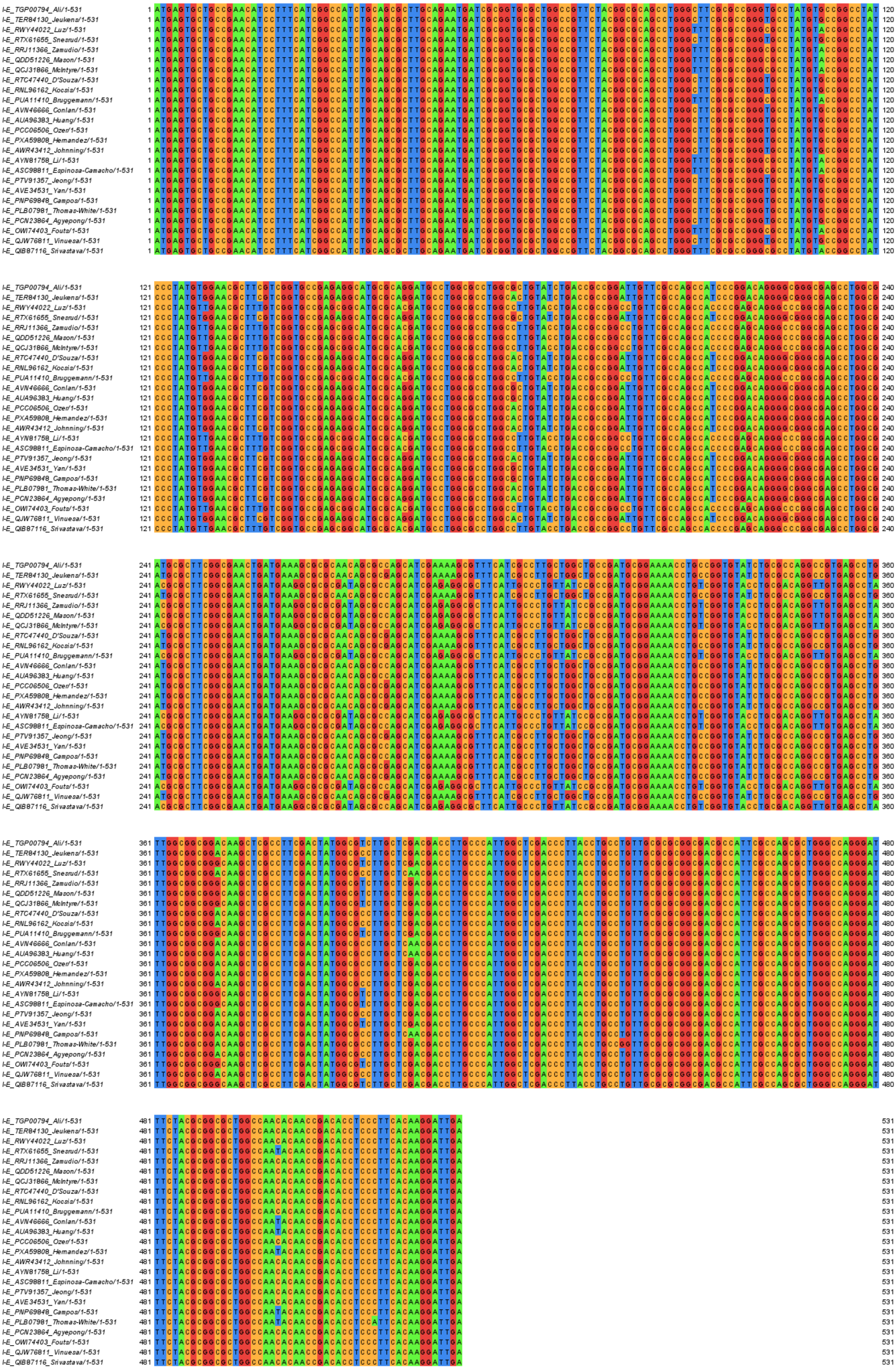
I-E_TGP00794_Ali/1-531
I-E_TER84130_Jeukens/1-531
I-E_RWY44022_Luz/1-531
I-E_RTX61655_Snesrud/1-531
I-E_RRJ11366_Zamudio/1-531
I-E_QDD51226_Mason/1-531
I-E_QCJ31866_McIntyre/1-531
I-E_RTC47440_D'Souza/1-531
I-E_RNL96162_Kocsis/1-531
I-E_PUA11410_Bruggemann/1-531
I-E_AVN46666_Conlan/1-531
I-E_AUA96383_Huang/1-531
I-E_PCC06506_Ozer/1-531
I-E_PXA59808_Hernandez/1-531
I-E_AWR43412_Johnning/1-531
I-E_AYN81758_Li/1-531
I-E_ASC98811_Espinosa-Camacho/1-531
I-E_PTV91357_Jeong/1-531
I-E_AVE34531_Yan/1-531
I-E_PNP69848_Campos/1-531
I-E_PLB07981_Thomas-White/1-531
I-E_PCN23864_Agyepong/1-531
I-E_OWI74403_Fouts/1-531
I-E_QJW76811_Vinuesa/1-531
I-E_QIB87116_Srivastava/1-531

G

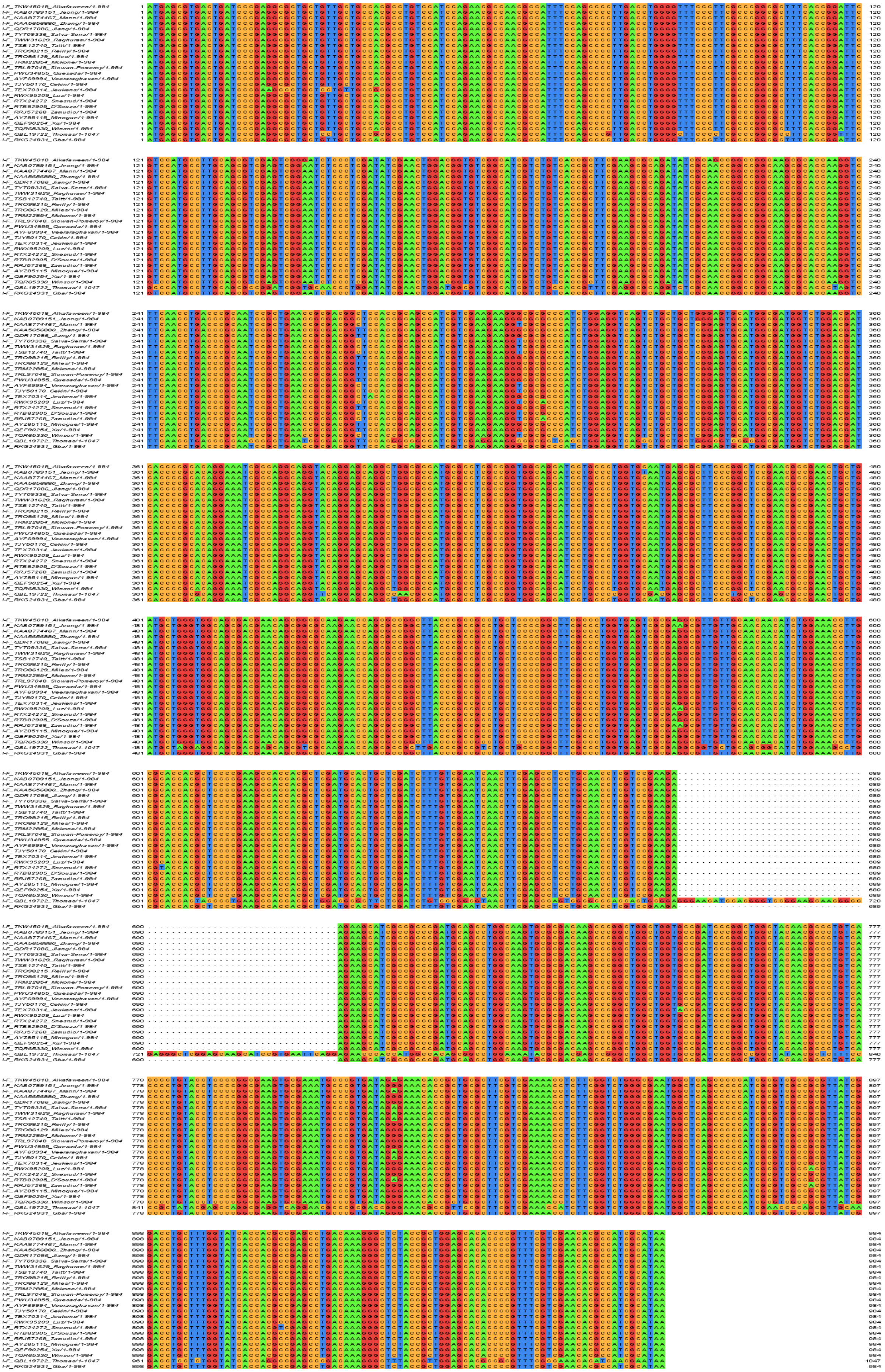

H

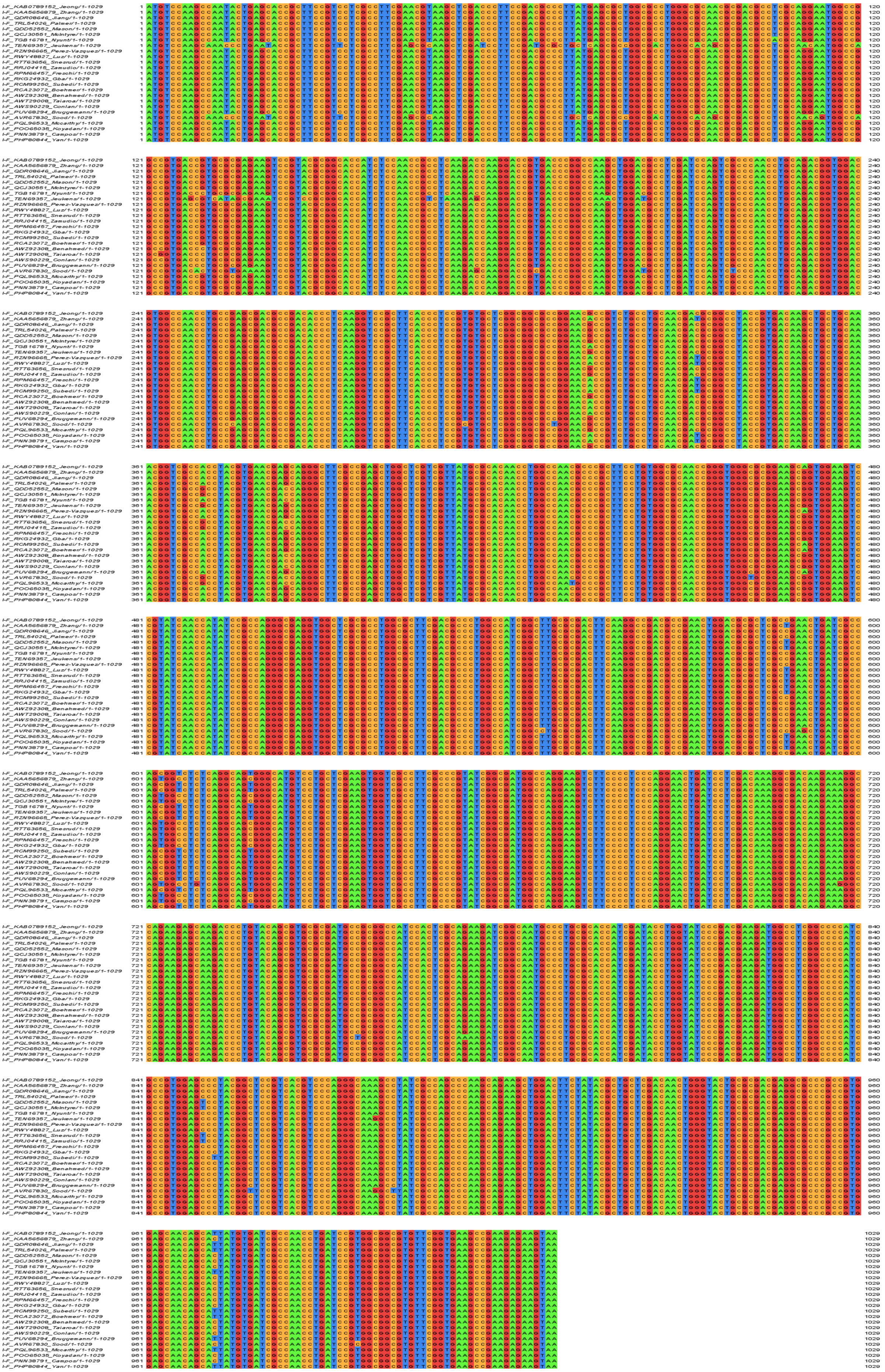

I

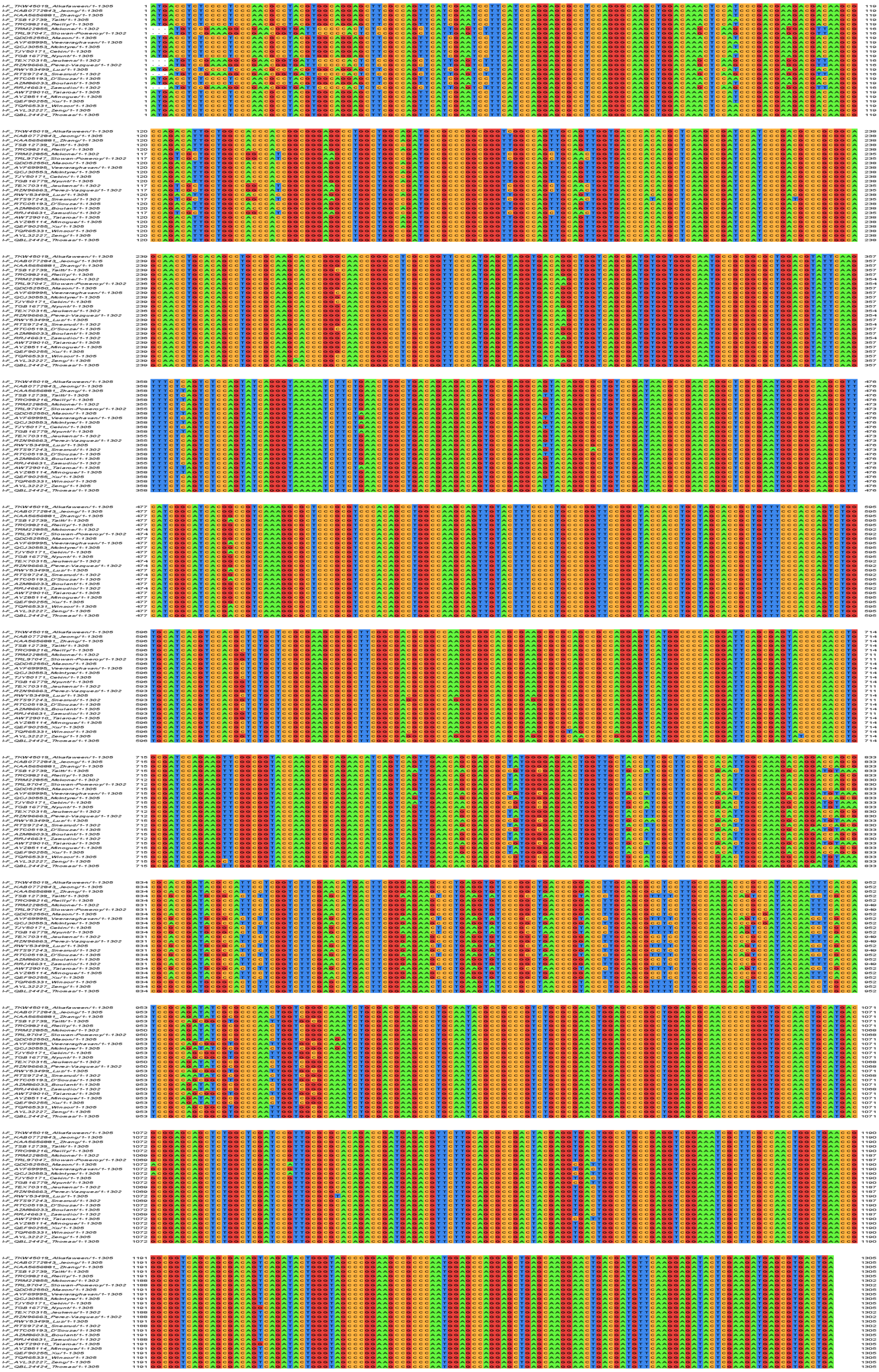

Supplement: Supplementary file 1 — Supplementary file1 (PDF 8911 KB) [file 253_2022_12144_MOESM1_ESM.pdf]
